# Supplementary material for: A novel conceptual approach to read-filtering in high-throughput amplicon sequencing studies
Source: Nucleic Acids Res. 2015 Nov 8;44(4):e40. doi: 10.1093/nar/gkv1113 (PMC4770208; doi:10.1093/nar/gkv1113)
Supplement: SUPPLEMENTARY DATA [file supp_44_4_e40__index.html]

A novel conceptual approach to read-filtering in high-throughput amplicon sequencing studies — SUPPLEMENTARY DATA 

# A novel conceptual approach to read-filtering in high-throughput amplicon sequencing studies

## SUPPLEMENTARY DATA

- SUPPLEMENTARY DATA
- SUPPLEMENTARY DATA
