# Supplementary material for: A novel conceptual approach to read-filtering in high-throughput amplicon sequencing studies
Source: Nucleic Acids Res. 2015 Nov 8;44(4):e40. doi: 10.1093/nar/gkv1113 (PMC4770208; doi:10.1093/nar/gkv1113)
Supplement: SUPPLEMENTARY DATA [file supp_gkv1113_SI_Oct_PROOFS.pdf]

A novel conceptual approach to read-filtering in high-throughput amplicon sequencing studies

Fernando Puente-Sánchez[1*], Jacobo Aguirre[1,2,3], Víctor Parro[1].

[1]Department of Molecular Evolution, Centro de Astrobiología (INTA-CSIC). Instituto Nacional de Técnica Aeroespacial, Ctra de Torrejón a Ajalvir km 4. 28850 Torrejón de Ardoz, Madrid, Spain.
[2]Centro Nacional de Biotecnología (CSIC). c/ Darwin 3, 28049 Madrid, Spain.
[3]Grupo Interdisciplinar de Sistemas Complejos (GISC). Madrid, Spain.

[*]Correspondence should be addressed to F.P.S. (fpusan@gmail.com)

Supplementary information

Supplementary Note 1. The Poisson binomial filtering algorithm.

Supplementary Note 2. Generation of taxonomic biases during filtration of a population of sequences.

Supplementary Note 3. Datasets used in this study.

Supplementary Note 4. Results on mock communities.

Supplementary Note 5. Results on environmental communities.

Supplementary Note 6. Random removal of reads does not explain the taxonomic biases generated by the different filtering methods.

Supplementary Note 7. Effect of the choice of quality-filtering method on the inference of ecological patterns.

Supplementary Note 8. Execution time of the Poisson binomial filtering algorithm and its Poisson approximation.

Supplementary Note 9. Theoretical and practical differences between the Poisson binomial filtering algorithm and USEARCH expected errors filtering

Supplementary Note 10. Software commands, parameters and computational resources.

Supplementary References.

Supplementary Note 1. The Poisson binomial filtering algorithm

SN1.1 Error probability distribution associated to a sequence

Let us suppose we have 1 sequence of length N nucleotides, each nucleotide with a potentially non-equal (and known) probability p_i of being erroneous and a probability $(1 - p_i)$ of being correct. We are interested in obtaining the probability of a sequence of having $j = 0, 1, 2, ..., N$ erroneous nucleotides. Statistically, our problem can be analysed as the probability distribution of the number of successes in a sequence of N independent yes/no experiments with success probabilities $p_1, p_2, ..., p_N$. This is equivalent to the sum S_N of N independent Bernoulli distributed random variables $X_1, X_2, ..., X_N$ such that

$$S_N = \sum_{i=1}^{N} X_i$$

where

$$P(X_i = j) = \begin{cases} 1 - p_i & \text{for } j = 0\,, \\ p_i & \text{for } j = 1\,, \\ 0 & \text{for } j > 1\,, \end{cases} \qquad \text{(SN1.1)}$$

and $P(X_i = j)$ stands for the probability of obtaining j errors in nucleotide i.

The stochastic variable S_N is known to follow a Poisson binomial distribution, from where the method proposed in this paper takes its name (see (Poisson, 1837) and (Cramér, 1999) for more information on this statistical distribution). The mean and variance of this distribution are

$$\begin{aligned} E[S_N] &= \sum_{i=1}^{N} p_i\,, \\ Var(S_N) &= \sum_{i=1}^{N} (1 - p_i) p_i\,. \end{aligned}$$

Following Eq. (SN1.1) and taking into account that all X_i are independent, the probability of the sequence of having zero errors is

$$P(S_N = X_1 + X_2 + ... + X_N = 0) = \prod_{i=1}^{N} (1 - p_i)\,,$$

the probability of the sequence of having one error is

$$P(S_N = 1) = \sum_{i=1}^{N} p_i \prod_{\substack{k=1 \\ k\neq i}}^{N} (1 - p_k)\,,$$

and the probability of the sequence of having j errors is

$$P(S_N = j) = \sum_{\sigma\in C(N,j)} \left(\prod_{x=\sigma_1}^{\sigma_j} p_x\right) \left(\prod_{y=\sigma_1^c}^{\sigma_{N-j}^c} (1 - p_y)\right) \qquad \text{(SN1.2)}$$

where σ is a map from $\{1, 2, ..., j\}$ to $E = \{1, 2, ..., N\}$ in a way that represents the combination of N elements taken j at a time without repetition, and satisfies $\sigma_r \leq \sigma_s$ for $r < s$. Note that σ^c is the complement of σ, i.e. $\sigma^c = \{1, 2, ..., N\} \setminus \sigma$. For example, if $N = 4$ and $j = 2$, $\sigma = \{\{1, 2\}, \{1, 3\}, \{1, 4\}, \{2, 3\}, \{2, 4\}, \{3, 4\}\}$ and $\sigma^c = \{\{3, 4\}, \{2, 4\}, \{2, 3\}, \{1, 4\}, \{1, 3\}, \{1, 2\}\}$.

SN1.2 Algorithm to obtain the error probability distribution of a sequence

While Eq. (SN1.2) is exact for all j, it becomes useless in practice for moderate values of j, as the number of elements in the summation of the equation is

$$\binom{N}{j} = \frac{N!}{j!(N-j)!},$$

which grows extremely fast with j. We explain here an algorithm inspired in (Butler & Stephens, 1993) that allows us to calculate $P(S_N = j)$ for all j in a simple and efficient way.

If we have two random variables Y and Z, each of them taking discrete values $0, 1, 2, \ldots$, the probability of the sum $Y + Z$ of taking value j is

$$P(Y + Z = j) = \sum_{i=0}^{j} P(Y = i)P(Z = j - i)\,. \qquad \text{(SN1.3)}$$

As we know the estimated probability p_i of each nucleotide of being erroneous, the basic idea of the algorithm is to calculate the error probability of the first two nucleotides of having j errors, add the third nucleotide to calculate the error probability of the first three nucleotides of having j errors, and continue recursively until we reach the N-th nucleotide and finally obtain the probability that the total sequence has j errors. When this is done for $j = 0, 1, 2, ..., N$ errors, we obtain the error probability distribution of the total sequence.

The algorithm results:

1. Obtain $P(X_1 = j)$ following Eq. (SN1.1). Let $U = X_1$.
2. For $i = 2, 3, ..., N$, the distribution is obtained by following (a-c) recursively.
   (a) Calculate $P(X_i = j)$ applying Eq. (SN1.1).
   (b) Calculate $P(Y + Z = j)$ applying Eq. (SN1.3), being $Y = U$ and $Z = X_i$. Note that all the terms $P(Y = i)$ and $P(Z = j - i)$ for $i = 0, ..., j$ needed in Eq. (SN1.3) were already calculated in previous steps of the algorithm.
   (c) Let $U = Y + Z$.
3. The probability for the sequence under study of having j errors, $P(S_N = j)$, is given by U when $i = N$.
4. The steps (1-3) must be repeated for $j = 0, 1, 2, ..., N$.

SN1.3 Filtering of sequences based on their error distribution

1. In practice, steps (1-3) of the algorithm presented in the former section must only be repeated up to $j = j_{max}$, where j_{max} is the lowest value of j that satisfies $\sum_{r=0}^{r=j} P(S_N = r) \geq \xi$, and $0 < \xi < 1$ is a confidence coefficient (in our case $\xi = 0.995$). Let the predicted maximum errors j_ξ be the number such that the sequence has a probability ξ of having less than j_ξ errors. It is obtained interpolating the accumulated error probability of the sequence between the values $r = j_{max} - 1$ and $r = j_{max}$ to obtain its exact value in $r = j_\xi$. A linear interpolation yields

$$j_\xi = j_{max} - 1 + \frac{\xi - \sum_{r=0}^{j_{max}-1} P(S_N = r)}{P(S_N = j_{max})} \,. \qquad \text{(SN1.4)}$$

2. Let j_{tol} be the maximum tolerable number of errors per sequence, that is, the maximum number of errors allowed for a correct clustering. In our case, j_{tol} was equal to 1% of the sequence length (i.e. $j_{tol} = 2$ for $N = 200$ nt or $j_{tol} = 2.5$ for $N = 250$ nt in the samples analysed in this work). The sequence under study is discarded if $j_\xi > j_{tol}$, and accepted as correct otherwise. At this moment, the calculation for this particular sequence is finished, and it is time to repeat the whole algorithm (subsections SN1.2 and SN1.3) for the rest of the sequences of the population.

Let us note that the default value of j_{tol} was chosen as 1% of the sequence length instead of a fixed integer number. We believe that the predicted number of errors alone is not a reliable estimator of sequence goodness as its interpretation depends on sequence size (e.g. the effect of having 2 errors is more critical in a 10 nt sequence than in a 200 nt sequence). We therefore propose to use the number of errors per base as a filtering parameter, for the sake of generality. Similarly, clustering is usually done based on relative sequence similarity (e.g. 97% similarity) instead of absolute Hamming distance. This fact results in the same choice of parameters having similar effects regardless of sequence length. Note, however, that all this could lead to non-integer values in the number of errors. We believe this is not a problem: the concept of predicted maximum errors j_ξ should be understood as a maximum bound and in a statistical context. In fact, the possibility of having non-integer error thresholds allows the user to fine tune the parameters in order to reach an optimal solution. Anyway, we provide the user with the possibility of using only integer error values (via the −−round flag in the moira.py script, or by selecting an integer error threshold, for example via the −−maxerrors parameter in the moira.py script).

Supplementary Figures SN1.1a,b show the error probability distribution and the accumulated error probability distribution respectively for a high quality sequence, an ambiguous sequence and a low quality sequence. The results obtained by the algorithm presented above (i.e. the Poisson binomial filtering method) are compared to those obtained with a Poisson approximation (see next subsection). **Supplementary Figure SN1.1c** shows a zoom of (**b**) to clarify the algorithm just presented.

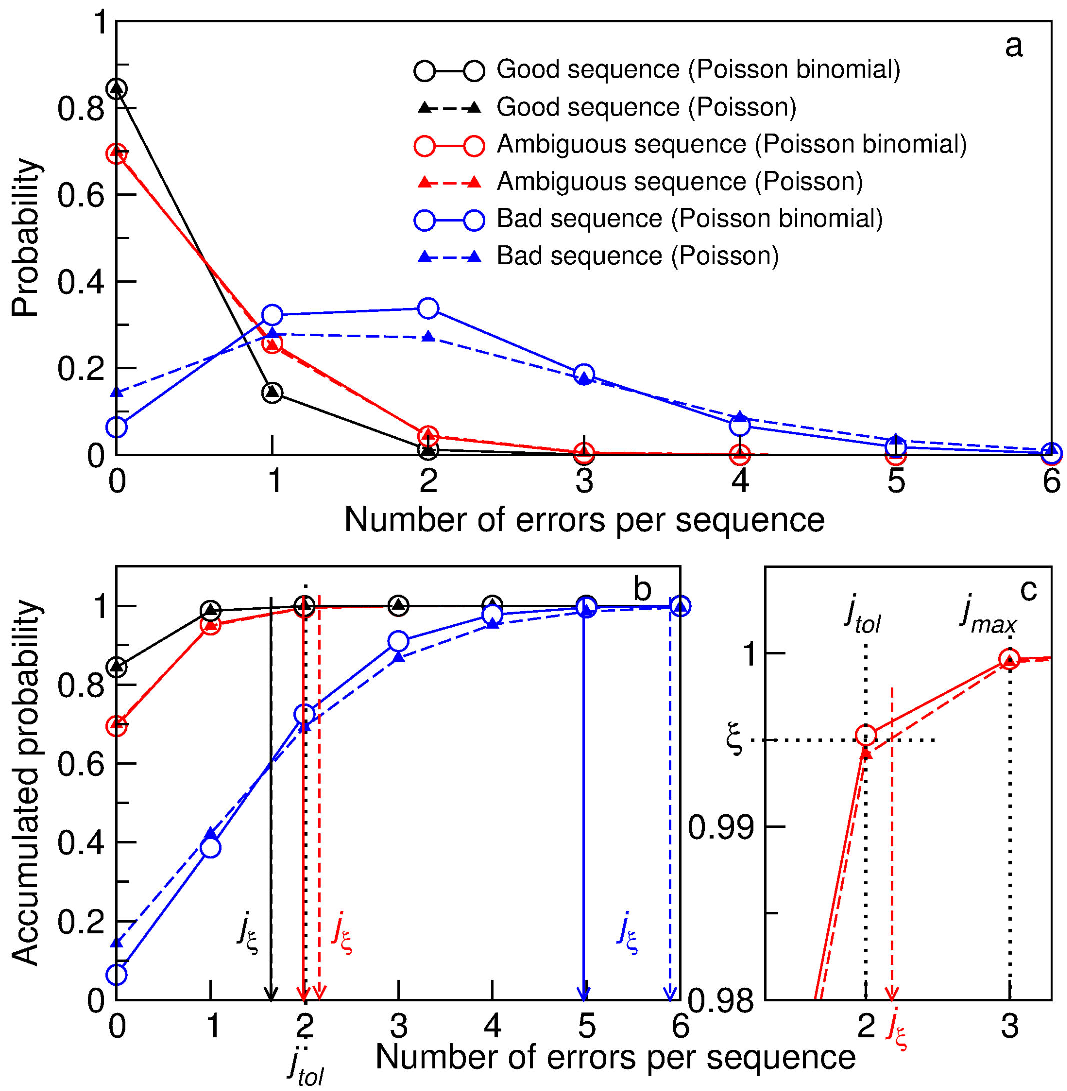

Figure SN1.1. Error probability distribution (**a**) and accumulated error probability distribution (**b**) for three particular sequences of N = 200 nucleotides. The Poisson binomial exact calculation (circles connected with solid lines) and the Poisson approximation (triangles connected with dashed lines) are plotted for a high quality sequence (black), an ambiguous sequence (red) and a low quality sequence (blue). The predicted maximum errors j_ξ stands for the 100ξ-th percentile of the error probability (i.e. a sequence has a probability ξ of having less than j_ξ errors). In (**c**) we plot a zoom of (**b**) where the calculation of j_ξ for the Poisson approximation of the ambiguous sequence is sketched (and where the difference between the Poisson binomial and its Poisson approximation is more evident). j_{max} is the lowest integer for which the accumulated probability of having j_{max} errors is greater than ξ. In our calculations, ξ = 0.995 and the maximum tolerable number of errors per sequence is j_{tol} = 2. Sequences with $j_\xi > j_{tol}$ are discarded in the filtering step.

SN1.4 A simple numerical example to clarify the Poisson binomial filtering algorithm

Let us numerically solve an especially simple case to clarify the applicability of the Poisson binomial filtering algorithm.

We have a short sequence of $N = 3$ nucleotides, whose error probabilities are $p_1 = p_2 = p_3 = 1/3$. They are all the same in this particular case to simplify the calculations, but in general they can be non-equal.

First, note that, following Eq. (SN1.1), $P(X_i = 0) = 2/3$, $P(X_i = 1) = 1/3$ and $P(X_i \geq 1) = 0$ for $i = 1, 2, 3$.

1. For $j = 0$, and applying Eq. (SN1.3), we obtain:

$$\begin{aligned}
P(X_1 + X_2 = 0) &= \sum_{i=0}^{j=0} P(X_1 = i)P(X_2 = j - i) = P(X_1 = 0)P(X_2 = 0) = 4/9\,,\\
P(X_1 + X_2 + X_3 = 0) &= \sum_{i=0}^{j=0} P(X_1 + X_2 = i)P(X_3 = j - i) = P(X_1 + X_2 = 0)P(X_3 = 0) = 8/27.
\end{aligned}$$

   Therefore, the probability for the sequence under study of having $j = 0$ errors is $P(S_3 = X_1 + X_2 + X_3 = 0) = 8/27$.

2. For $j = 1$, we obtain:

$$\begin{aligned}
P(X_1 + X_2 = 1) &= \sum_{i=0}^{j=1} P(X_1 = i)P(X_2 = j - i)\\
&= P(X_1 = 0)P(X_2 = 1) + P(X_1 = 1)P(X_2 = 0) = 4/9\,,\\
P(X_1 + X_2 + X_3 = 1) &= \sum_{i=0}^{j=1} P(X_1 + X_2 = i)P(X_3 = j - i)\\
&= P(X_1 + X_2 = 0)P(X_3 = 1) + P(X_1 + X_2 = 1)P(X_3 = 0) = 4/9.
\end{aligned}$$

   Therefore, the probability for the sequence under study of having $j = 1$ errors is $P(S_3 = X_1 + X_2 + X_3 = 1) = 4/9$.

3. For $j = 2$, we obtain:

$$\begin{aligned}
P(X_1 + X_2 = 2) &= \sum_{i=0}^{j=2} P(X_1 = i)P(X_2 = j - i) = P(X_1 = 0)P(X_2 = 2)\\
&+ P(X_1 = 1)P(X_2 = 1) + P(X_1 = 0)P(X_2 = 2) = 1/9\,,\\
P(X_1 + X_2 + X_3 = 2) &= \sum_{i=0}^{j=2} P(X_1 + X_2 = i)P(X_3 = j - i) = P(X_1 + X_2 = 0)P(X_3 = 2)\\
&+ P(X_1 + X_2 = 1)P(X_3 = 1) + P(X_1 + X_2 = 2)P(X_3 = 0) = 2/9.
\end{aligned}$$

Therefore, the probability for the sequence under study of having $j = 2$ errors is $P(S_3 = X_1 + X_2 + X_3 = 2) = 2/9$.

4. For $j = 3$, we obtain:

$$\begin{aligned}
P(X_1 + X_2 = 3) &= \sum_{i=0}^{j=3} P(X_1 = i)P(X_2 = j - i) = P(X_1 = 0)P(X_2 = 3) \\
&+ P(X_1 = 1)P(X_2 = 2) + P(X_1 = 2)P(X_2 = 1) + P(X_1 = 3)P(X_2 = 0) = 0\,, \\
P(X_1 + X_2 + X_3 = 3) &= \sum_{i=0}^{j=3} P(X_1 + X_2 = i)P(X_3 = j - i) = P(X_1 + X_2 = 0)P(X_3 = 3) \\
&+ P(X_1 + X_2 = 1)P(X_3 = 2) + P(X_1 + X_2 = 2)P(X_3 = 1) \\
&+ P(X_1 + X_2 = 3)P(X_3 = 0) = 1/27.
\end{aligned}$$

Therefore, the probability for the sequence under study of having $j = 3$ errors is $P(S_3 = X_1 + X_2 + X_3 = 3) = 1/27$.

In summary, we have that the sequence of 3 nucleotides of error probabilities $p_1 = p_2 = p_3 = 1/3$ has a probability 8/27 of having 0 errors, probability 4/9 of having 1 error, probability 2/9 of having 2 errors and probability 1/27 of having 3 errors.

Let us see now if it would be accepted or discarded according to the Poisson binomial filtering algorithm. Following Eq. (SN1.4) and taking into account that $j_{max} = 3$ and $\xi = 0.995$, we obtain $j_\xi = 2.865$. In case $j_{tol} = 2$, the algorithm would discard this sequence.

Finally, let us note that the binomial distribution is a special case of the Poisson binomial distribution, when all probabilities are the same, that is, when $p_1 = p_2 = \cdots = p_N$. Therefore, the calculations shown in this example could be checked with the explicit probability mass function of the binomial distribution (Cramér, 1999).

SN1.5 Approximate calculation of the error probability distribution of a sequence

In the former subsections we presented the Poisson binomial filtering (PBF) method to calculate the exact distribution of the error probability of a sequence, provided the error probabilities p_i of each of its nucleotides. The Poisson binomial filtering algorithm is fast and computationally simple. Here, we compare it with several approximations that can be used.

As our problem can be faced as the sum of N binomial distributions of probabilities p_i and number of trials $n = 1$, it can be approximated to a Poisson distribution as far as N is high and $p_i \ll 1$. The probability mass function of X is given by

$$f(j;\lambda) = P(S_N = j) = \frac{\lambda^j \exp(-\lambda)}{j!} \tag{SN1.5}$$

where $P(S_N = j)$ is the probability of a sequence of having j errors, and the mean value and variance of the Poisson distribution, λ, is obtained as

$$\lambda = \sum_{i=1}^{N} p_i\,. \tag{SN1.6}$$

The Le Cam's theorem affirms that the sum has approximately a Poisson distribution and the inequality

$$\sum_{j=0}^{\infty} \left| P(S_N = j) - \frac{\lambda^j \exp(-\lambda)}{j!} \right| < 2 \sum_{i=1}^{N} p_i^2 \tag{SN1.7}$$

bounds the approximation error in terms of the total variation distance between the exact solution and the approximation. The Poisson approximations following Eqs. (SN1.5) and (SN1.6) are plotted in **Supplementary Figure SN1.1** in triangles joined by dashed lines.

We can see that the accuracy of the Poisson approximation decreases for high values of p_i, that is, for sequences with low-quality bases. Therefore, the exact method and the Poisson approximation could lead to differences in the acceptance or rejection of sequences for j_{tol} sufficiently large and $j_\xi \sim j_{tol}$, i.e. for ambiguous sequences.

Note that the difference between the results obtained by the Poisson binomial filtering method and its Poisson approximation could be relevant in real cases. **Supplementary Figure SN1.2** shows the relationship between both methods for the sequences present in the Even1P dataset (see **Supplementary Note 3**). The Poisson binomial filtering discarded 11% less sequences than its Poisson approximation, which is remarkable considering that the target of the method presented here is to improve the precision of classifications as much as possible. But, what makes these 11% of sequences to be correctly accepted by the PBF method and wrongly rejected by the

Poisson approximation? In typical cases, we have found that this different precision is due to the existence of a unique nucleotide with a low quality score, preventing the satisfaction of the hypothesis $p_i \ll 1$ cited above. Let us show a simple example to clarify this point. A sequence of N=200 nucleotides, one of them with Q = 10 and the rest with Q = 30, would lead to a bound for the difference between the Poisson binomial distribution and its Poisson approximation obtained from the Le Cam's theorem of $2\sum_{i=1}^{N} p_i^2 = 0.0204$ (applying the equivalence $p_i = 10^{\frac{Q}{-10}}$). This value is 50 times larger than the bound obtained for the same sequence but where all nucleotides show the same quality Q = 30 (and where $2\sum_{i=1}^{N} p_i^2 = 4 \times 10^{-4}$, a very low value that makes the Poisson approximation a valid assumption). In fact, a large amount of real cases obtained from Even1P dataset that were accepted by the PBF method and wrongly rejected by its Poisson approximation gave rise to differences of one or even two orders of magnitude in the Le Cam's bound.

In summary, the precision of the Poisson approximation is extremely dependent on the existence of a few (even one) bad-quality nucleotides. This fact leads in those cases to values of j_ξ obtained by the Poisson binomial filtering and its Poisson approxi-

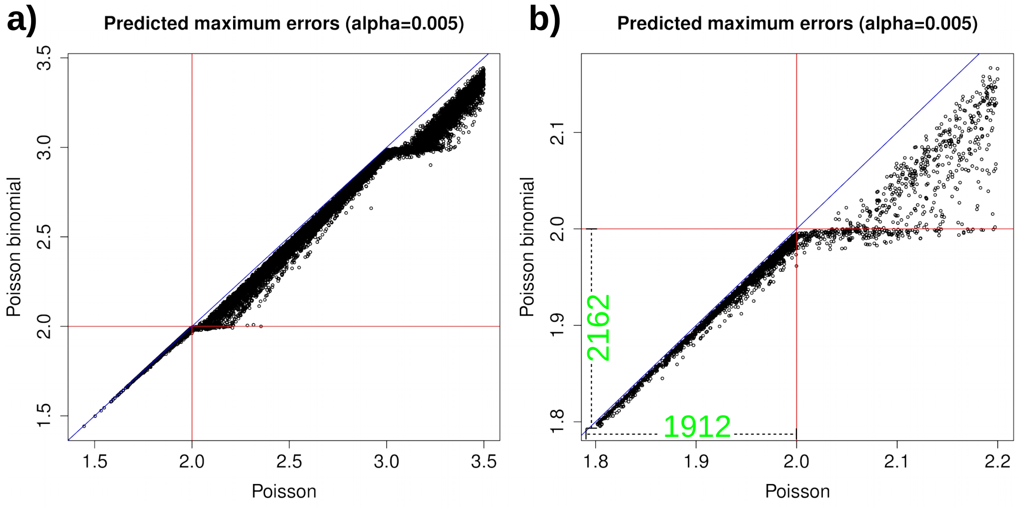

Figure SN1.2. Relationship between the Poisson binomial filtering method and its Poisson approximation for the sequences present in the Even1P dataset. a) High and intermediate quality sequences. b) Zoom showing high quality sequences and our error cutoff of 2 predicted maximum errors per sequence (1% of sequence length). Numbers in green: sequences accepted by the Poisson binomial filtering (y axis) and its Poisson approximation (x axis). PBF discarded 11% less sequences than its Poisson approximation.

mation sufficiently different to influence substantially the number of sequences that are accepted or not.

Other approximations of higher precision than the Poisson approximation, but of a more complex implementation, such as the Kolmogorov-type approximations or the ones obtained using the Pearson family of distributions, are also available (Butler & Stephens, 1993). However, the accuracy, simplicity and speed of the exact method make it in general a better option than the approximative methods, as the latter will be either less precise or even more complex to implement.

Supplementary Note 2. Generation of taxonomic biases during filtration of a population of sequences

SN2.1 Calculation of the statistical properties of a filtered population of sequences

Let us suppose that we have M sequences belonging to k different taxa. In a process of filtering we eliminate $m < M$ sequences chosen randomly from the total population in a way that all sequences are equally probable to be erased. We name $n_i(m)$ the number of sequences of taxon $i = 1, ..., k$ that remain in the population after removing m sequences, and

$$p_i(m) = \frac{n_i(m)}{M - m}$$

the fraction of such remaining population that belongs to taxon i. In this calculation, we are interested in the statistical properties (i.e., mean and variance) of the fraction $p_i(m)$ of sequences of each taxon i over the total number of remaining sequences when the number of removed sequences m grows.

The hypergeometric distribution applies to sampling without replacement from a finite population whose elements can be classified into two mutually exclusive categories (in our case, each taxon i and the rest of the population). Therefore, the stochastic variable

$$X_i = n_i(0) - n_i(m)$$

that represents the number of sequences removed from taxon i when m sequences are randomly chosen from the total population follows a hypergeometric distribution of population size M, number of initial success states in the population $n_i(m = 0)$ and number of draws m. In consequence, the probability mass function of X_i is given by

$$P(x) = \frac{\binom{n_i(0)}{x}\binom{M - n_i(0)}{m - x}}{\binom{M}{m}}$$

where $P(x)$ is the probability of erasing exactly x sequences from taxon i after eliminating m sequences in the total population. Its mean and variance are, respectively and for every taxon i,

$$\begin{aligned} E[X_i] &= \frac{n_i(0)m}{M}, \\ Var(X_i) &= \frac{(M - n_i(0))n_i(0)(M - m)m}{M^2(M - 1)}. \end{aligned}$$

As mentioned above, we are interested in the fraction of the remaining total population that belongs to taxon i after removing m sequences, that is, $p_i(m) = n_i(m)/(M-m)$. The stochastic variable $Y_i = p_i(m)$ verifies

$$Y_i = p_i(m) = \frac{n_i(m)}{M-m} = \frac{n_i(0) - X_i}{M-m} \,,$$

and its statistical properties are

$$\begin{aligned} E[Y_i] &= E[p_i(m)] = E\left[\frac{n_i(0) - X_i}{M-m}\right] = \frac{n_i(0)}{M-m} - \frac{n_i(0)m}{M(M-m)} \\ &= \frac{n_i(0)}{M} = p_i(0)\,, && \text{(SN2.1)} \\ Var(Y_i) &= Var(p_i(m)) = Var\left(\frac{n_i(0) - X_i}{M-m}\right) = \frac{Var(X_i)}{(M-m)^2} = \frac{n_i(0)(M - n_i(0))}{M^2(M-1)}\frac{m}{M-m} \\ &= \frac{p_i(0)(1 - p_i(0))}{M-1}\frac{m}{M-m}\,. && \text{(SN2.2)} \end{aligned}$$

Equation (SN2.1) yields that the mean value $E[p_i(m)] = p_i(0)$ and therefore is independent of the number of removed sequences m. On the contrary, its variance grows monotonically with m:

$$Var(p_i(m)) = \sigma^2(p_i(m)) \propto \frac{m}{M-m}\,.$$

In summary, we observe that the filtering process of a total population leads to fractions of each taxon that are maintained in average but grow in variance when the number of removed sequences m increases. This leads to the following general statement: the more sequences are erased, the larger error we will be committing when measuring the composition of the total population.

Finally, note that the term $M-1$ in the denominator of Eq. (SN2.2) moderates the influence of m in the generation of taxonomic bias (see **Supplementary Note 6** for empirical evidence). A greater source of taxonomic bias during sequence filtering is described in the next subsection.

SN2.2 Taxonomic bias is inherent to raw reads in 454 and Illumina sequencing platforms

Most of the discussion on taxonomic bias in massive sequencing projects has focused on the coverage bias (i.e. some fragments getting a larger number of amplicons than others) caused by PCR amplification (Polz & Cavanaugh, 1998; Huber *et al.*, 2009; Kumar *et al.*, 2011; Ross *et al.*, 2013; Klindworth *et al.*, 2013). However, it is generally assumed that, after sequencing a pool of DNA molecules, the length and quality distributions of the obtained reads are independent of their taxonomic origin. If that were not to happen, the length trimming and quality filtering processes would result in the under-representation of the taxa with smaller average length and quality, generating a bias in the retrieved community composition.

We have found evidences of this problem occurring in the 454 datasets analysed for this work. After taxonomically classifying all the raw reads from the 454 Even3T library with mothur, we found out that the reads classified into the genus *Staphylococcus* (the most abundant taxon in the sample) were significantly shorter than the reads classified as *Streptococcus* (the second most abundant taxon in the sample) (**Supplementary Figure SN2.1a,b**). We also found that the *Staphylococcus* reads had more expected errors per base than the *Streptococcus* reads (**Supplementary Figure SN2.1c**). This is not surprising: since in the 454 platform the read quality drops at the end of the reads (Edgar, 2013), the longer reads are expected to have a higher average quality. Our pipeline included a pre-filtering step in which reads were trimmed to a fixed length of 250 nt, and reads shorter than 250 nt were discarded. Because of the differences in the read length distributions, 11.7 % of the *Staphylococcus* reads were discarded during this step, in contrast to only 7.8% of the *Streptococcus* reads. This produced a 4.4% (since 92.2/88.3=1.044) artificial enrichment of *Streptococcus* versus *Staphylococcus* in the pre-filtered dataset. Pre-filtering also amplified the differences in quality between the *Staphylococcus* and the *Streptococcus* reads. Due to the relationship between position and quality in 454 reads, trimming to a fixed length will leave only high quality nucleotides in the longer reads, but may leave low quality nucleotides at the end of the shorter ones. Differences in length distribution will thus result in differences in quality distribution after trimming (**Supplementary Figure SN2.1c,d**). Even if the pre-filtering step considerably reduced the average expected errors per base, it also caused a significant increase in the taxonomic bias. Performing quality filtering with our default cutoff of 0.01 errors per base would have discarded 59.6% of the *Staphylococcus* reads but only 37.0% of the *Streptococcus* reads, resulting in a 56.2% artificial enrichment of *Streptococcus* versus *Staphylococcus* in the filtered dataset. Note that this comes in addition to the previous enrichment caused by the length trimming step.

Length and quality biases were also detected for the IonTorrent platform, a fact that has been described elsewhere (Salipante *et al.*, 2014). For example, filtering with our 200nt length and 0.01 errors per base cutoffs removed 89.46% of the *Bacteroides* (the

most abundant genus in the sample) reads from the IonTorrent Even2P, but 94.56% of the *Streptococcus* (second most abundant genus in the sample) reads, resulting in a 192% artificial enrichment of *Bacteroides* versus *Streptococcus*. A similar problem, albeit to a lesser extent, was found in reads generated using the Illumina MiSeq platform. In this case, reads from different taxa showed different quality distributions. When quality-filtering raw reads from MiSeq Even1M library, we found out that 63.03% of the reads classified as *Staphylococcus* (the most abundant genus in the sample) had 0.01 errors per base or more, while 68.37% of the reads classified as *Acinetobacter* had 0.01 errors per base or more. Filtering with those parameters caused a 16.9% artificial enrichment of *Staphylococcus* versus *Acinetobacter*.

These biases are likely originated during base/quality calling: for instance, 454 reads show a systematic decrease in quality after homopolymer regions (Brockman *et al.*, 2008), which will penalise the taxa with longer homopolymer stretches on its 16S gene. We propose the incorporation of a simple step into the filtering pipeline that substantially minimises this problem. It is based on the assumption of the fact that, in spite of differences in quality, identical sequences should have the same origin, as it is unlikely that two biologically unrelated sequences became identical due to sequencing errors. Thus, identical sequences were collapsed, and the representative with the highest overall quality was used to decide whether the whole group was discarded or allowed into the filtered dataset. In practice, this mitigates the effect of biases in quality distribution as even low abundance sequences are expected to have a high quality representative. Our solution rendered similar quality distributions for the different taxa, even after length trimming (**Supplementary Figure SN2.1e,f**), and significantly lower taxonomic biases than other filtering approaches, especially for 454 data (**Figure 2c**). Every method that relies on quality scores for sequence filtering will be affected by this source of bias. We therefore propose the approach described above as a general solution to this problem, since its simplicity makes it very easy to integrate into any filtering pipeline.

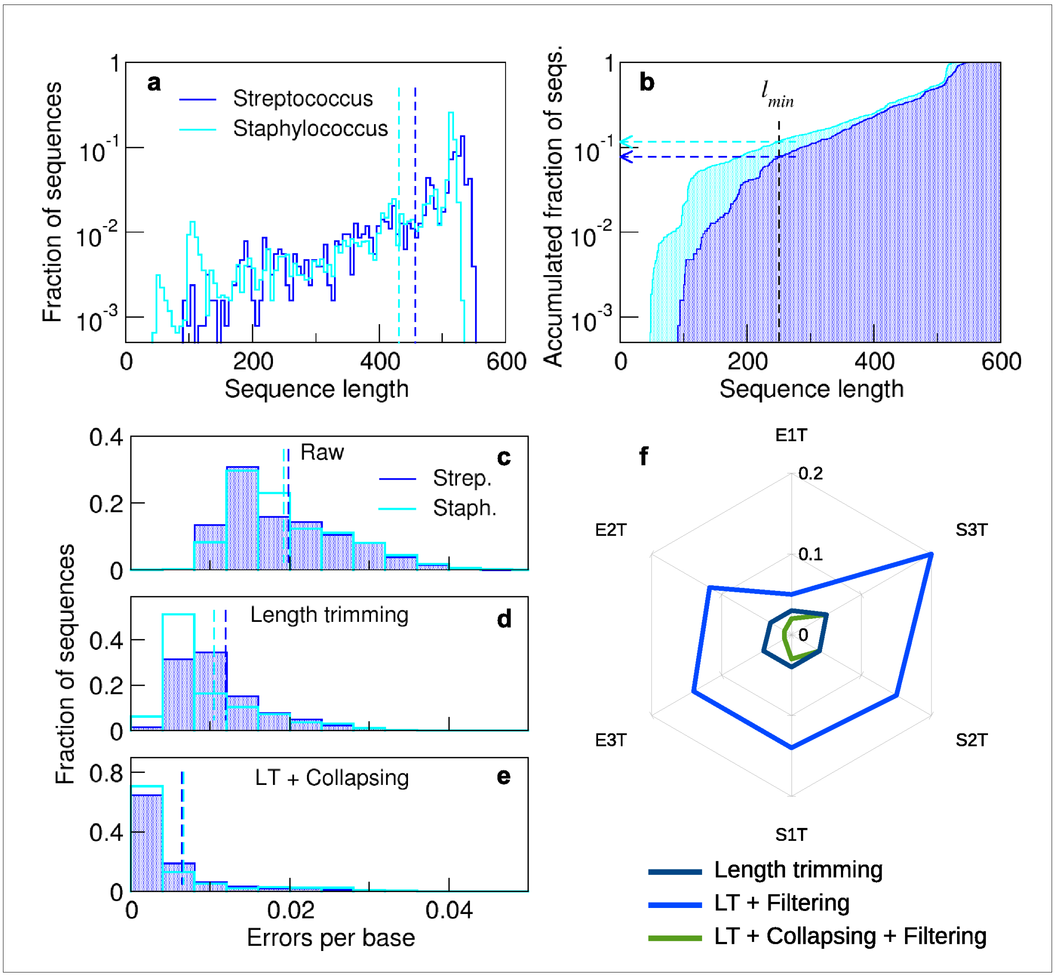

Figure SN2.1. Addressing the taxonomic bias generated during the pre-processing and quality filtering of raw sequences. (**a, b**) Raw reads from *Streptococcus* and *Staphylococcus*, the two most abundant genera in sample Even3T, show different length distributions. The dashed vertical lines in (**a**) indicate the average read lengths. The arrows in (**b**) indicate the fraction of reads from each taxon removed after discarding sequences shorter than l_{min} = 250 nt. (**c, d, e**) Errors per base distributions of *Streptococcus* and *Staphylococcus* reads in the (**c**) raw dataset, (**d**) after trimming the reads to 250 nt and discarding the ones shorter than the cut-off, and (**e**) after collapsing the trimmed reads. The dashed lines indicate average errors per base. Note that length trimming substantially increases the difference between the *Streptococcus* and *Staphylococcus* error distributions (**d**) when compared to that of the raw reads (**c**). Filtering at this point would cause a 56.2% overrepresentation of *Streptococcus* versus *Staphylococcus.* Collapsing identical reads prior to filtering solves this problem (**e**), reducing the overrepresentation to 1%. (**f**) Compositional bias generated during the pre-processing and filtering of the six 454 mock community samples, measured as the Bray-Curtis dissimilarity between the raw and the processed read communities. This shows that results in (**c, d, e**) can be generalised to all the taxa present in all the samples.

Supplementary Note 3. Datasets used in this study

Table SN3.1. Dataset accession numbers and abbreviations

This table shows details on the 16S datasets used in this study. NCBI Short Read Archive accession numbers are provided, unless otherwise specified. The 454 even mock community 1 was taken from the example in mothur's 454 SOP webpage (http://www.mothur.org/wiki/454_SOP). The Illumina mock communities were taken from Bokulich *et al.*, 2013 and Edgar, 2013. For Illumina datasets, the number of pairs is provided. The IonTorrent environmental communities were taken from mothur's SOP webpage (http://www.mothur.org/wiki/Ion_Torrent_sequence_analysis_using_Mothur).

Dataset name	Abbreviation	Community type	Accession	Platform	Reads/Pairs
454 HMP Even Mock Community 1 (mothur SOP)	Even1T/E1T	Mock, even	(mothur's 454 SOP)	454 GS FLX Titanium	8,969
454 HMP Even Mock Community 2	Even2T/E2T	Mock, even	SRR072220	454 GS FLX Titanium	24,922
454 HMP Even Mock Community 3	Even3T/E3T	Mock, even	SRR072239	454 GS FLX Titanium	23,850
454 HMP Staggered Mock Community 1	Stag1T/S1T	Mock, staggered	SRR072221	454 GS FLX Titanium	23,049
454 HMP Staggered Mock Community 2	Stag2T/S1T	Mock, staggered	SRR072223	454 GS FLX Titanium	46,541
454 HMP Staggered Mock Community 3	Stag3T/S3T	Mock, staggered	SRR072237	454 GS FLX Titanium	20,449
Illumina HMP Even Mock Community 1	Even1M/E1M	Mock, even	(Bokulich *et al.*, 2013)	Illumina MiSeq	1,520,374*
Illumina HMP Even Mock Community 2	Even2M/E2M	Mock, even	(Bokulich *et al.*, 2013)	Illumina MiSeq	1,644,911*
Illumina HMP Staggered Mock Community 1	Stag1M/S1M	Mock, staggered	(Bokulich *et al.*, 2013)	Illumina MiSeq	1,857,075*
Illumina HMP Staggered Mock Community 2	Stag2M/S2M	Mock, staggered	(Bokulich *et al.*, 2013)	Illumina MiSeq	2,576,656*
IonTorrent HMP Even Mock Community 1	Even1P/E1P	Mock, even	SRR1204732	IonTorrent PGM	4,313,746*
IonTorrent HMP Even Mock Community 2	Even2P/E2P	Mock, even	SRR1204734	IonTorrent PGM	4,261,813*
Bacterial Sequences from Saliva	HumanOral	Environmental	SRR823350	454 GS FLX Titanium	5,057
Cryoconite sediments	IceMetagenome	Environmental	SRR1142358	454 GS FLX Titanium	12,159
Storr's Lake Microbial Mat	MicrobialMat	Environmental	SRR1015086	454 GS FLX Titanium	22,642
Pig gut metagenome	PigGut	Environmental	SRR1174681	454 GS FLX Titanium	17,321
Bacterial diversity in agricultural soils	SoilMetagenome	Environmental	SRR1119147	454 GS FLX Titanium	6,660
Wastewater Treatment Plant in Jonkowo	WasteWater	Environmental	SRR1019213	454 GS FLX Titanium	6,031
Concrete Corrosion Biofilm	ConcreteCorrosion	Environmental	SRR869416	Illumina MiSeq	24,291
Gull fecal bacterial community	GullFecal	Environmental	SRR1036650	Illumina MiSeq	110,158*
Human gut metagenome	HumanGut	Environmental	SRR1212611	Illumina MiSeq	35,577
Negative control from a soil metagenome project	LabWater	Environmental	SRR1371022	Illumina MiSeq	1,058
Mouse metagenome isolated from cecum of mouse	MouseGut	Environmental	SRR651466	Illumina MiSeq	144,051*
Microbial community diversity in petroleum reservoirs	PetroleumReservoir	Environmental	SRR1295596	Illumina MiSeq	115,443*
mothur SOP IonOct49	Ion49	Environmental	(mothur's IonTorrent SOP)	IonTorrent PGM	5,513
mothur SOP IonOct51	Ion51	Environmental	(mothur's IonTorrent SOP)	IonTorrent PGM	4,783
mothur SOP IonOct57	Ion57	Environmental	(mothur's IonTorrent SOP)	IonTorrent PGM	5,214
mothur SOP IonOct58	Ion57	Environmental	(mothur's IonTorrent SOP)	IonTorrent PGM	5,584
mothur SOP IonOct61	Ion61	Environmental	(mothur's IonTorrent SOP)	IonTorrent PGM	5,603
mothur SOP IonOct62	Ion62	Environmental	(mothur's IonTorrent SOP)	IonTorrent PGM	6,123
mothur SOP IonOct64	Ion64	Environmental	(mothur's IonTorrent SOP)	IonTorrent PGM	5,064
mothur SOP IonOct68	Ion68	Environmental	(mothur's IonTorrent SOP)	IonTorrent PGM	5,244
mothur SOP IonOct71	Ion71	Environmental	(mothur's IonTorrent SOP)	IonTorrent PGM	5,709
mothur SOP IonOct72	Ion72	Environmental	(mothur's IonTorrent SOP)	IonTorrent PGM	5,354
mothur SOP IonOct76	Ion76	Environmental	(mothur's IonTorrent SOP)	IonTorrent PGM	5,423
mothur SOP IonOct78	Ion78	Environmental	(mothur's IonTorrent SOP)	IonTorrent PGM	5,113
mothur SOP IonOct79	Ion79	Environmental	(mothur's IonTorrent SOP)	IonTorrent PGM	6,214

* When a dataset contained more than 40,000 reads, all analyses were performed over a random subsample of 40,000 raw reads. For each of those datasets, an additional file containing the names of the randomly selected sequences is provided as a **Supplementary resource.**

Supplementary Note 4. Results on mock communities

SN4.1. Poisson binomial filtering accurately discriminates between good and erroneous sequences

When applying our default cut-off of 1% errors allowed per sequence, our algorithm accurately classified 96% of the mock community sequences from the Even1M dataset (**Figure 1d**). 3% of the sequences were incorrectly discarded while, remarkably, only 1% of the sequences were incorrectly retained. Moreover, most of those incorrectly retained sequences had only 3 true errors (1.2% errors per sequence), meaning that they would likely cluster correctly when applying the standard 3% OTU distance cut-off. The rest of the Illumina datasets rendered similar results. The accuracy of our method was slightly lower for the 454 and IonTorrent datasets, but it nevertheless resulted in a minimum of 88% (for 454) and 79% (for IonTorrent) correctly classified sequences. (**Supplementary Figure SN4.1**).

SN4.2. Performance of the different filtering methods on the mock community datasets

Publicly available datasets from even and staggered mock communities from the Human Microbiome Project (Haas *et al.*, 2011) were filtered with PBF, mothur, USEARCH and QIIME (**Figure 2, Supplementary Tables SN4.2, SN4.4**). These artificial communities contain known amounts of 16S rRNA gene copies from 20 different bacterial organisms. The fact that both the qualitative and quantitative composition of the samples are known beforehand allowed us to thoroughly compare the effects of the different filtering methods in terms of OTU accuracy, alpha diversity and community composition. OTU accuracy was defined as the maximum similarity of its representative sequence to the 16S sequences of the microorganisms used to build the mock community, as previously described in Edgar (2013). We were also interested in determining how the different filtering processes affected the observed community composition. The taxonomic bias in community composition caused by any given filtering method was calculated as the Bray-Curtis dissimilarity between the raw and the filtered datasets, after taxonomically classifying their reads down to the genus level.

In the even datasets, which contain the same number of 16S rRNA gene copies for each organism, all methods resulted in more than 20 OTUs after clustering. This was not surprising, since contaminations, PCR errors and sequencing errors were expected to inflate the observed diversity. In the staggered communities, in which the number of 16S rRNA gene copies varied by several orders of magnitude between the different organisms, the observed diversity was generally lower, due to some species being present at very low abundances. The total number of reported OTUs greatly varied between filtering methods, with Poisson binomial filtering consistently resulting in values that were the closest to the true diversity of the samples.

PBF also produced the highest proportion of accurate OTUs in all the 16S mock datasets for both sequencing platforms, while minimizing the number of singletons and spurious OTUs retrieved (**Figure 2a,b**). In the 454 and IonTorrent datasets it also discarded the smallest number of reads and resulted in the smallest taxonomic bias (**Figure 2c,d**). In the Illumina datasets QIIME retrieved a larger number of reads, while both QIIME and mothur caused smaller taxonomic biases than our method. (**Figure 2c,d - Illumina**). However, we

believe that this was the result of a too shallow filtering by mothur and QIIME, since both methods produced a remarkably lower proportion of accurate OTUs and a larger number of OTUs and singletons (**Figure 2a,b - Illumina**). QIIME produced an especially high number of spurious OTUs, a fact that has also been discussed elsewhere (Edgar, 2013). Their pipeline (Bokulich *et al.,* 2013) deals with this problem by applying a post-hoc OTU size cut-off at the cost of sensitivity. Nonetheless, our results show that, even after the removal of singletons from the QIIME-filtered dataset, their number of OTUs would exceed that of the dataset filtered with our method, including singletons (**Supplementary Table SN4.4**).

The two filtering algorithms included in the USEARCH suite showed an intermediate performance in terms of the number and accuracy of the OTUs retrieved for both the 454 and Illumina platforms. Quality trimming yielded the smallest number of reads and resulted in the highest taxonomic bias, which supports the idea that over-stringent filtering may lead to undesirable effects. In the IonTorrent datasets, USEARCH filtering (as recommended in http://www.brmicrobiome.org/#!16sprofilingpipeline/cuhd) performed below Poisson binomial filtering for all the studied bechmarks (**Figure 2 - IonTorrent**). Finally, the mothur implementation of the PyroNoise algorithm (Quince *et al.,* 2009) showed lower OTU accuracy than the other methods tested for filtering 454 reads. It has been previously described that the denoising process can introduce minor alterations in the original reads (Gaspar & Thomas, 2013), a phenomenon that might explain these results. It must be noted that, albeit a pipeline for filtering IonTorrent reads with PyroNoise has been described, the publicly available IonTorrent mock community datasets were only available in Fastq format (Stephen Salipante, personal communication), which precluded the use of flowgram denoising algorithms. However, this limitation was not present for the environmental datasets, and a comparison of quality filtering algorithms for IonTorrent datasets that includes PyroNoise can therefore be found in **Supplementary Figure SN5.3**.

Table SN4.1. Taxonomic composition of the 454 16S mock community datasets

This table gives the taxonomic composition of the raw datasets from the 16S mock communities sequenced with the 454 GS FLX Titanium platform, classified at the genus level with mothur's *classify.seqs* command.

	Even1T	Even2T	Even3T	Stag1T	Stag2T	Stag3T
Acinetobacter	7.3875%	2.3724%	2.0786%	0.1262%	0.1550%	0.1072%
Actinomyces	1.6250%	1.8452%	1.6869%	0.0049%	0.0049%	0.0000%
Aeromonas	0.0000%	0.0000%	0.0000%	0.0000%	0.0025%	0.0000%
Anaerobacter	0.0000%	0.0088%	0.0000%	0.0000%	0.0049%	0.0000%
Azorhizophilus	0.0000%	0.0000%	0.0000%	0.0097%	0.0025%	0.0000%
Bacillus	0.7250%	0.6151%	0.5623%	0.5290%	0.4551%	0.4512%
Bacteroides	19.3125%	8.0090%	8.2915%	0.0388%	0.0517%	0.0282%
Clostridium_sensu_stricto	5.3875%	2.9918%	3.0788%	2.2227%	2.5411%	1.6695%
Corynebacterium	0.0000%	0.0000%	0.0000%	0.0000%	0.0025%	0.0000%
Deinococcus	7.6875%	34.2149%	33.9770%	0.1359%	0.0418%	0.0677%
Devosia	0.0000%	0.0000%	0.0000%	0.0000%	0.0000%	0.0056%
Enterococcus	0.8500%	0.6634%	0.6222%	0.0971%	0.0861%	0.3610%
Escherichia_Shigella	0.8000%	0.4964%	0.3180%	2.9069%	2.9962%	0.6317%
Helicobacter	1.3750%	0.1142%	0.0784%	0.0049%	0.0049%	0.0113%
Lactobacillus	0.6250%	0.3515%	0.3365%	0.0194%	0.0271%	0.0451%
Lactovum	0.0000%	0.0044%	0.0000%	0.0000%	0.0000%	0.0000%
Listeria	1.6625%	1.3751%	1.5025%	0.0971%	0.0984%	0.1072%
Methanobrevibacter	0.0000%	0.0308%	0.0184%	0.1407%	0.2608%	0.1184%
Mycobacterium	0.0000%	0.0000%	0.0000%	0.0097%	0.0098%	0.0226%
Neisseria	5.1000%	2.1615%	2.1985%	0.0922%	0.1451%	0.0564%
Prevotella	0.0125%	0.0000%	0.0000%	0.0000%	0.0000%	0.0000%
Propionibacterium	4.9000%	3.1280%	2.7469%	0.0874%	0.1009%	0.0226%
Pseudomonas	1.2500%	0.9797%	0.9540%	0.8396%	0.7109%	0.4230%
Rhodobacter	0.3250%	0.5623%	0.5899%	5.1393%	4.0639%	4.5065%
Salmonella	0.0000%	0.0000%	0.0046%	0.0097%	0.0394%	0.0169%
Staphylococcus	31.2500%	34.6147%	35.1293%	66.4418%	65.4104%	69.4078%
Stenotrophomonas	0.0000%	0.0000%	0.0000%	0.0049%	0.0000%	0.0000%
Streptococcus	9.7250%	5.4609%	0.0000%	21.0376%	22.7842%	21.9402%
Sulfitobacter	0.0000%	0.0000%	5.8257%	0.0049%	0.0000%	0.0000%

Figure SN4.1. Poisson binomial filtering accurately discriminates between good and erroneous sequences.

Comparison between the maximum predicted errors j_ξ calculated by the Poisson binomial algorithm and the true number of errors for all sequences from the 454 and Illumina MiSeq mock community dataset. Dots represent unique sequences. True mock community sequences are plotted in blue, contaminant sequences are plotted in gray, and chimeric sequences are plotted in red. The blue background represents sequence abundance. Red lines indicate our error cutoff of 2.5 errors per sequence (j_{tol}, 2 errors per sequence for the IonTorrent datasets). The percentage of true mock community sequences present on each quadrant is also indicated. The graph is plotted in logarithmic scale (the 0 in the x-axis is added for clarity).

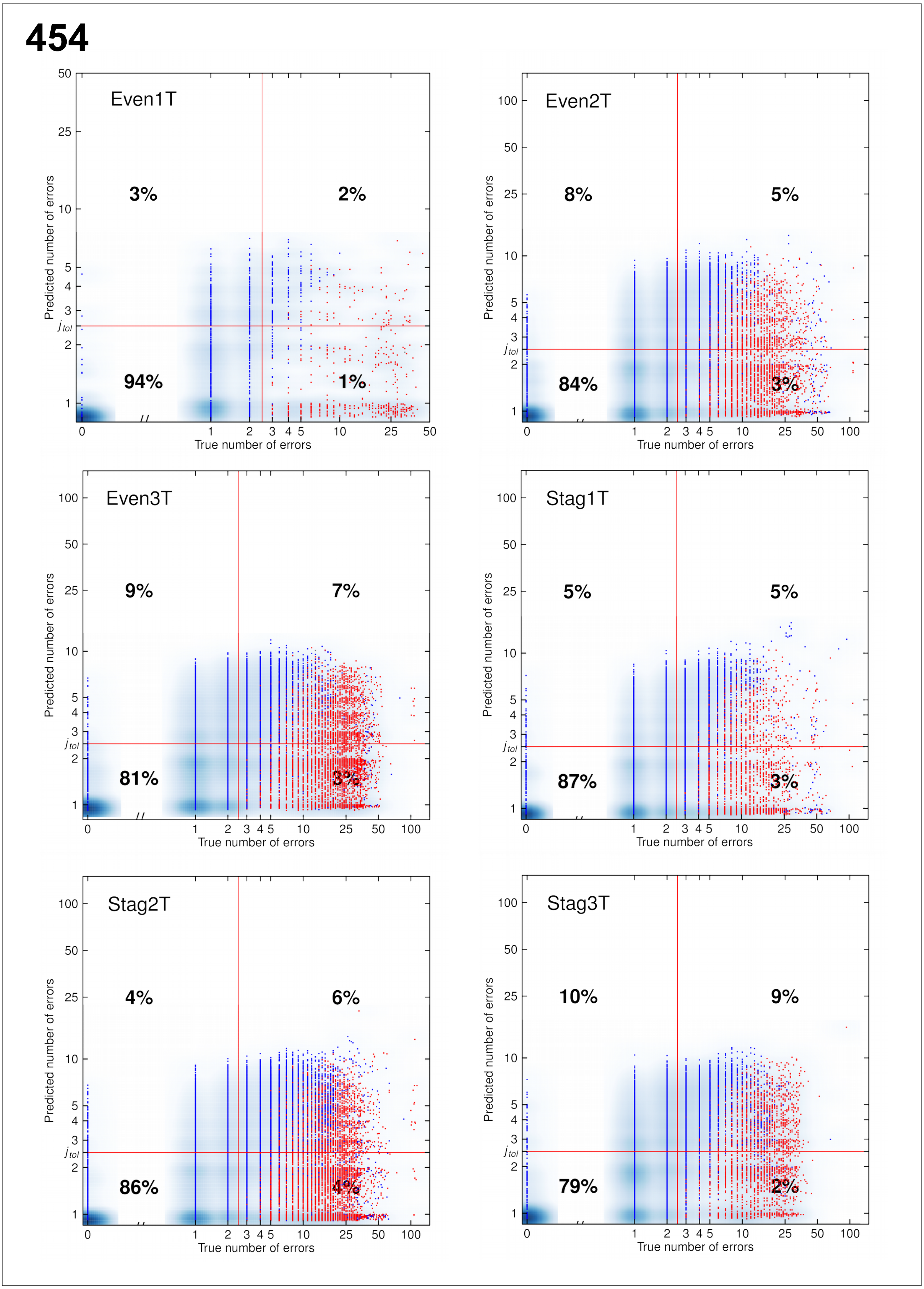
454
Even1T
3%
2%
94%
1%
Even2T
8%
5%
84%
3%
Even3T
9%
7%
81%
3%
Stag1T
5%
5%
87%
3%
Stag2T
4%
6%
86%
4%
Stag3T
10%
9%
79%
2%
Predicted number of errors
True number of errors
j_{tol}

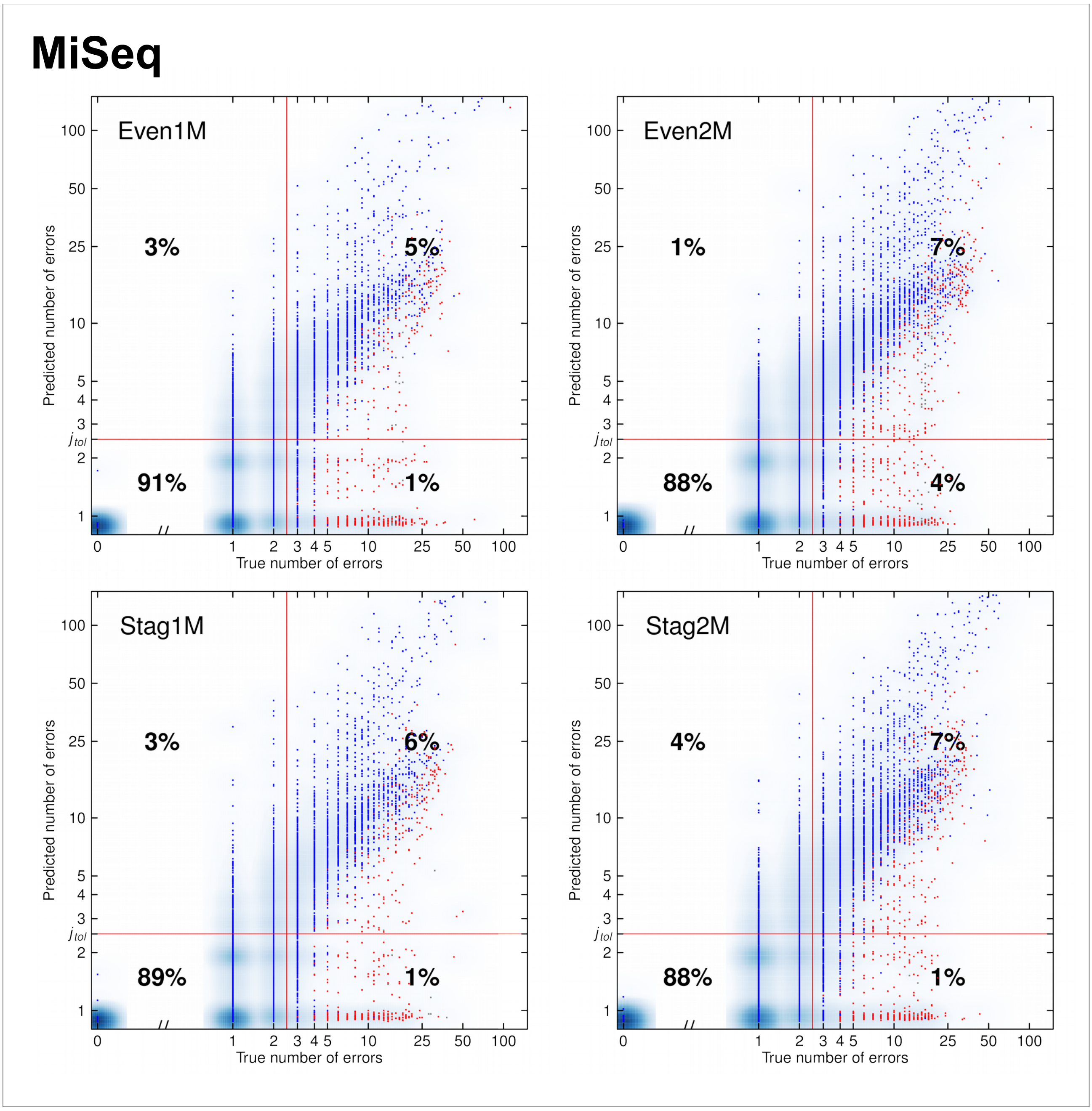
MiSeq
Even1M
Even2M
Stag1M
Stag2M
3%
5%
91%
1%
1%
7%
88%
4%
3%
6%
89%
1%
4%
7%
88%
1%
Predicted number of errors
True number of errors
j_{tol}
100
50
25
10
5
4
3
2
1
0
1
2
3
4
5
10
25
50
100

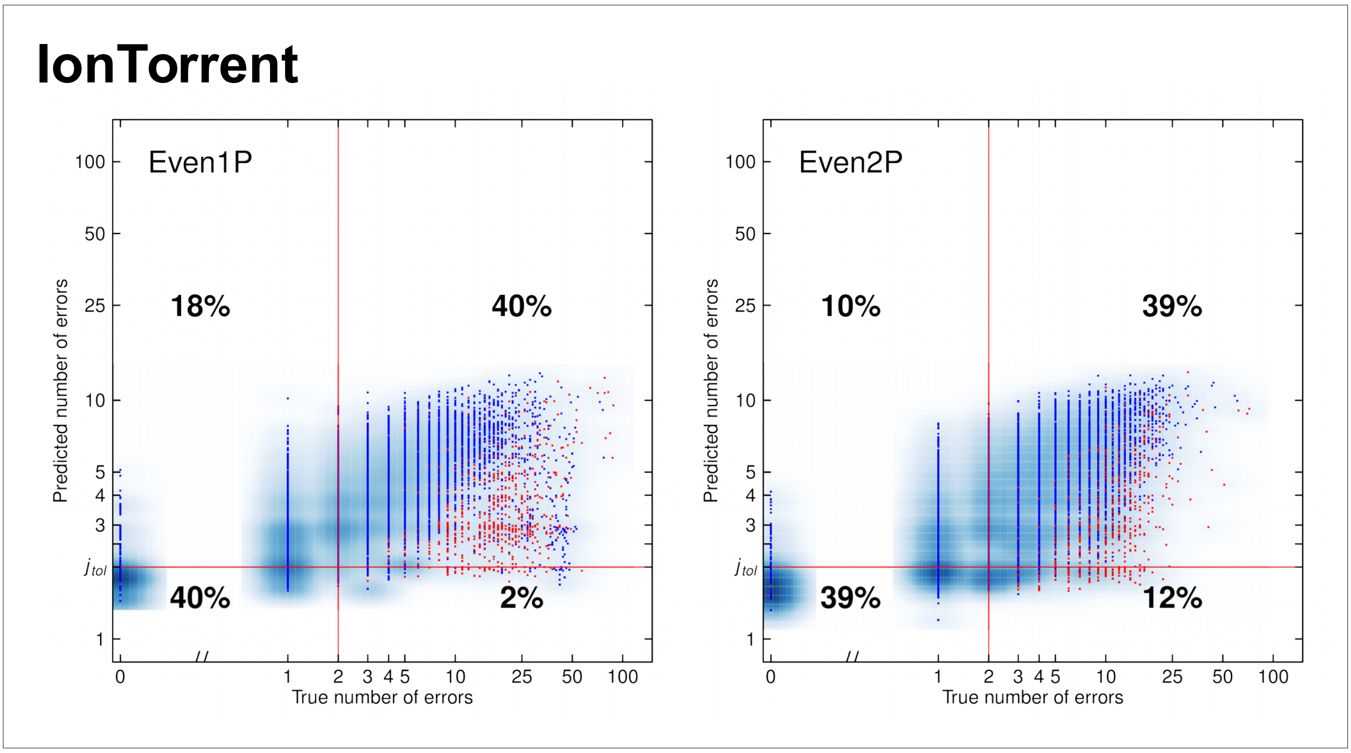

Table SN4.2. Results on the 454 mock communities

This table shows the results obtained after quality filtering the six 454 mock communities with different filtering methods. For each method, the “NoSUB” table shows the results obtained directly after filtering, while the “SUB” table shows the results obtained after performing 100 independent random sub-samplings of the filtered reads. For each sample, the number of reads kept by the most stringent method was chosen as the sub-sampling size for the rest of the methods. Abbreviations: std, standard deviation; Coverage, Good's coverage; TaxBias, taxonomic bias (see methods).

Even1T

Poisson binomial filtering

NoSUB

OTUs	Singletons	Coverage	TaxBias	#Reads passing filters
25	6	0.9992	0.0293	7421

Perfect	Good	Noisy	Contaminant	Other
16	0	3	4	2

SUB_6071

OTUs	OTUs_std	Singletons	Singletons_std	Coverage	Coverage_std
23.93	1.06	5.2	1.06	0.9991	0.0002

Perfect	Good	Noisy	Contaminant	Other
16.12	0	2.79	3.12	1.9

PyroNoise

NoSUB

OTUs	Singletons	Coverage	TaxBias	#Reads passing filters
26	8	0.9987	0.0432	6071

Perfect	Good	Noisy	Contaminant	Other
0	0	18	0	8

USEARCH expected errors

NoSUB

OTUs	Singletons	Coverage	TaxBias	#Reads passing filters
25	6	0.9991	0.0498	6915

Perfect	Good	Noisy	Contaminant	Other
16	0	3	4	2

SUB_6071

OTUs	OTUs_std	Singletons	Singletons_std	Coverage	Coverage_std
24.26	1.05	5.56	1.06	0.9991	0.0002

Perfect	Good	Noisy	Contaminant	Other
16.12	0	2.68	3.53	1.93

Quality-trimming

NoSUB

OTUs	Singletons	Coverage	TaxBias	#Reads passing filters
24	5	0.9992	0.0826	6320

Perfect	Good	Noisy	Contaminant	Other
17	0	1	3	3

SUB_6071

OTUs	OTUs_std	Singletons	Singletons_std	Coverage	Coverage_std
24.05	0.64	5.05	0.64	0.9992	0.0001

Perfect	Good	Noisy	Contaminant	Other
17	0	1.18	2.91	2.96

Even2T

Poisson binomial filtering

NoSUB

OTUs	Singletons	Coverage	TaxBias	#Reads passing filters
34	11	0.9994	0.0271	16963

Perfect	Good	Noisy	Contaminant	Other
17	1	3	0	13

SUB_7437

OTUs	OTUs_std	Singletons	Singletons_std	Coverage	Coverage_std
26.78	2	6.1	2.1	0.9992	0.0003

Perfect	Good	Noisy	Contaminant	Other
17.5	1.06	1.27	0	6.95

PyroNoise

NoSUB

OTUs	Singletons	Coverage	TaxBias	#Reads passing filters
42	19	0.9985	0.0702	12951

Perfect	Good	Noisy	Contaminant	Other
17	1	1	1	22

SUB_7437

OTUs	OTUs_std	Singletons	Singletons_std	Coverage	Coverage_std
32.81	2.42	12.54	2.5	0.9983	0.0003

Perfect	Good	Noisy	Contaminant	Other
17.73	0.98	0.38	0.48	13.24

USEARCH expected errors

NoSUB

OTUs	Singletons	Coverage	TaxBias	#Reads passing filters
37	15	0.9984	0.1047	9251

Perfect	Good	Noisy	Contaminant	Other
18	1	2	0	16

SUB_7437

OTUs	OTUs_std	Singletons	Singletons_std	Coverage	Coverage_std
33.9	1.46	12.45	1.51	0.9983	0.0002

Perfect	Good	Noisy	Contaminant	Other
17.75	1	1.8	0	13.35

Quality-trimming

NoSUB

OTUs	Singletons	Coverage	TaxBias	#Reads passing filters
26	6	0.9992	0.1281	7437

Perfect	Good	Noisy	Contaminant	Other
16	2	2	0	6

Even3T

Poisson binomial filtering

NoSUB

OTUs	Singletons	Coverage	TaxBias	#Reads passing filters
22	3	0.9998	0.0445	15123

Perfect	Good	Noisy	Contaminant	Other
17	1	2	0	2

SUB_5876

OTUs	OTUs_std	Singletons	Singletons_std	Coverage	Coverage_std
20.14	0.92	1.19	0.9	0.9998	0.0002

Perfect	Good	Noisy	Contaminant	Other
17.69	1	0.73	0	0.72

PyroNoise

NoSUB

OTUs	Singletons	Coverage	TaxBias	#Reads passing filters
27	5	0.9993	0.0471	12951

Perfect	Good	Noisy	Contaminant	Other
17	1	1	0	8

SUB_5876

OTUs	OTUs_std	Singletons	Singletons_std	Coverage	Coverage_std
21.94	0.93	2.17	1.01	0.9996	0.0002

Perfect	Good	Noisy	Contaminant	Other
17.81	1	0.84	0	2.29

USEARCH expected errors

NoSUB

OTUs	Singletons	Coverage	TaxBias	#Reads passing filters
27	5	0.9993	0.13	7610

Perfect	Good	Noisy	Contaminant	Other
17	1	1	0	8

SUB_5876

OTUs	OTUs_std	Singletons	Singletons_std	Coverage	Coverage_std
25.76	1.02	4.34	1.12	0.9993	0.0002

Perfect	Good	Noisy	Contaminant	Other
17.2	1	0.6	0	6.96

Quality-trimming

NoSUB

OTUs	Singletons	Coverage	TaxBias	#Reads passing filters
26	8	0.9986	0.1418	5876

Perfect	Good	Noisy	Contaminant	Other
16	3	1	0	6

Stag1T

Poisson binomial filtering

NoSUB

OTUs	Singletons	Coverage	TaxBias	#Reads passing filters
23	9	0.9994	0.0398	15189

Perfect	Good	Noisy	Contaminant	Other
15	1	3	0	4

SUB_7866

OTUs	OTUs_std	Singletons	Singletons_std	Coverage	Coverage_std
18.49	1.57	5.78	1.8143	0.9993	0.0002

Perfect	Good	Noisy	Contaminant	Other
14.05	0.49	1.81	0	2.14

PyroNoise

NoSUB

OTUs	Singletons	Coverage	TaxBias	#Reads passing filters
27	12	0.9989	0.0856	10750

Perfect	Good	Noisy	Contaminant	Other
13	1	4	0	9

SUB_7866

OTUs	OTUs_std	Singletons	Singletons_std	Coverage	Coverage_std
23.59	1.7	8.87	1.68	0.9989	0.0002

Perfect	Good	Noisy	Contaminant	Other
13.26	0.72	3.18	0	6.4

USEARCH expected errors

NoSUB

OTUs	Singletons	Coverage	TaxBias	#Reads passing filters
25	10	0.9990	0.1233	10177

Perfect	Good	Noisy	Contaminant	Other
15	2	4	0	4

SUB_7866

OTUs	OTUs_std	Singletons	Singletons_std	Coverage	Coverage_std
22.63	1.41	8.77	1.56	0.9989	0.0002

Perfect	Good	Noisy	Contaminant	Other
14.17	1.53	3.85	0	2.95

Quality-trimming

NoSUB

OTUs	Singletons	Coverage	TaxBias	#Reads passing filters
25	10	0.9987	0.2081	7866

Perfect	Good	Noisy	Contaminant	Other
15	1	3	0	6

Stag2T

Poisson binomial filtering					
NoSUB					
OTUs	Singletons	Coverage	TaxBias	#Reads passing filters	
27	8	0.9997	0.0408	30660	
Perfect	Good	Noisy	Contaminant	Other	
15	0	2	0	10	
SUB_15567					
OTUs	OTUs_std	Singletons	Singletons_std	Coverage	Coverage_std
22.09	1.6	6.02	1.75	0.9996	0.0001
Perfect	Good	Noisy	Contaminant	Other	
14	0	2.05	0	6.04	

PyroNoise					
NoSUB					
OTUs	Singletons	Coverage	TaxBias	#Reads passing filters	
30	12	0.9995	0.0725	21922	
Perfect	Good	Noisy	Contaminant	Other	
14	2	3	0	11	
SUB_15567					
OTUs	OTUs_std	Singletons	Singletons_std	Coverage	Coverage_std
26.44	1.61	9.36	1.55	0.9994	0.0001
Perfect	Good	Noisy	Contaminant	Other	
13.26	1.67	2.93	0	8.58	

USEARCH expected errors					
NoSUB					
OTUs	Singletons	Coverage	TaxBias	#Reads passing filters	
28	9	0.9995	0.1392	19899	
Perfect	Good	Noisy	Contaminant	Other	
15	0	3	0	10	
SUB_15567					
OTUs	OTUs_std	Singletons	Singletons_std	Coverage	Coverage_std
25.97	1.16	8.24	1.39	0.9995	0.0001
Perfect	Good	Noisy	Contaminant	Other	
14.46	0	3.02	0	8.49	

Quality-trimming				
NoSUB				
OTUs	Singletons	Coverage	TaxBias	#Reads passing filters
32	10	0.9994	0.2263	15567
Perfect	Good	Noisy	Contaminant	Other
14	1	4	0	13

Stag3T

Poisson binomial filtering

NoSUB

OTUs	Singletons	Coverage	TaxBias	#Reads passing filters
25	10	0.9991	0.0545	11014

Perfect	Good	Noisy	Contaminant	Other
10	2	2	1	10

SUB_3690

OTUs	OTUs_std	Singletons	Singletons_std	Coverage	Coverage_std
16.76	1.7	5.45	1.9	0.9985	0.0005

Perfect	Good	Noisy	Contaminant	Other
9.75	1.05	1.06	0.35	4.55

PyroNoise

NoSUB

OTUs	Singletons	Coverage	TaxBias	#Reads passing filters
23	6	0.9992	0.0989	7327

Perfect	Good	Noisy	Contaminant	Other
11	2	2	1	7

SUB_3690

OTUs	OTUs_std	Singletons	Singletons_std	Coverage	Coverage_std
19.44	1.53	5.15	1.73	0.9986	0.0005

Perfect	Good	Noisy	Contaminant	Other
10.92	1.22	1.69	0.49	5.12

USEARCH expected errors

NoSUB

OTUs	Singletons	Coverage	TaxBias	#Reads passing filters
26	10	0.9981	0.1864	5288

Perfect	Good	Noisy	Contaminant	Other
10	3	1	1	11

SUB_3690

OTUs	OTUs_std	Singletons	Singletons_std	Coverage	Coverage_std
22.49	1.57	8.67	1.79	0.9977	0.0005

Perfect	Good	Noisy	Contaminant	Other
9.73	2.42	1.33	0.61	8.4

Quality-trimming

NoSUB

OTUs	Singletons	Coverage	TaxBias	#Reads passing filters
23	8	0.9978	0.3376	3690

Perfect	Good	Noisy	Contaminant	Other
9	2	1	1	10

Table SN4.3. Taxonomic composition of the Illumina 16S mock community datasets

This table gives the taxonomic composition of the raw datasets from the 16S mock communities sequenced with the Illumina MiSeq platform, classified at the genus level with mothur's *classify.seqs* command. The taxonomic composition from both the assembled paired-end reads (labeled with the suffix "P") and the forward single reads (labeled with the suffix "S") is provided.

	Even1M_P	Even1M_S	Even2M_P	Even2M_S	Stag1M_P	Stag1M_S	Stag2M_P	Stag2M_S
Acinetobacter	21.7594%	21.3406%	17.9154%	18.3935%	0.8911%	0.9059%	0.8478%	0.8718%
Actinomyces	1.1290%	1.1484%	1.3095%	1.3039%	0.0134%	0.0163%	0.0055%	0.0056%
Aeromonas	0.0000%	0.0000%	0.0026%	0.0000%	0.0000%	0.0000%	0.0000%	0.0000%
Alkanindiges	0.0000%	0.0000%	0.0000%	0.0027%	0.0000%	0.0000%	0.0000%	0.0028%
Azomonas	0.0000%	0.0027%	0.0026%	0.0027%	0.0000%	0.0000%	0.0000%	0.0000%
Azotobacter	0.0000%	0.0027%	0.0000%	0.0000%	0.0000%	0.0000%	0.0000%	0.0000%
Bacillus	0.0000%	0.0080%	0.0079%	0.0054%	0.0080%	0.0109%	0.0055%	0.0139%
Bacteroides	3.7790%	4.1118%	4.2584%	4.2724%	0.0348%	0.0408%	0.0436%	0.0472%
Clostridium_sensu_stricto	2.4043%	2.2941%	2.4447%	2.4669%	1.5734%	1.5560%	1.7037%	1.7298%
Cronobacter	0.0000%	0.0000%	0.0000%	0.0000%	0.0000%	0.0000%	0.0027%	0.0000%
Deinococcus	6.0161%	6.1570%	7.3974%	7.5743%	0.0696%	0.0707%	0.0518%	0.0500%
Devosia	0.0000%	0.0000%	0.0000%	0.0000%	0.0107%	0.0109%	0.0000%	0.0000%
Dorea	0.0052%	0.0000%	0.0000%	0.0000%	0.0000%	0.0000%	0.0000%	0.0000%
Enterococcus	0.9252%	0.8914%	0.8897%	0.7970%	0.0054%	0.0054%	0.0082%	0.0083%
Escherichia_Shigella	0.5018%	0.3962%	0.3485%	0.4283%	1.9908%	2.3585%	2.3443%	2.6433%
Helicobacter	2.3469%	2.4200%	2.5397%	2.6025%	0.1606%	0.1632%	0.2017%	0.2027%
Lactobacillus	0.1829%	0.1579%	0.3802%	0.2223%	0.0080%	0.0027%	0.0273%	0.0194%
Listeria	2.3730%	2.3771%	2.4209%	2.4480%	0.0776%	0.0843%	0.1363%	0.1472%
Methanobrevibacter	0.4809%	0.4872%	0.4435%	0.4581%	6.9867%	7.0947%	6.8175%	6.9414%
Neisseria	1.9757%	1.8069%	2.0434%	2.0766%	0.1552%	0.1578%	0.0927%	0.0944%
Phenylobacterium	0.0000%	0.0000%	0.0000%	0.0000%	0.0000%	0.0000%	0.0027%	0.0028%
Propionibacterium	0.0157%	0.0054%	0.0158%	0.0163%	0.0000%	0.0000%	0.0000%	0.0000%
Pseudomonas	2.2371%	2.0211%	2.2124%	2.0467%	1.2898%	1.1480%	1.3357%	1.2356%
Rhodobacter	0.9774%	0.9931%	0.8844%	0.9136%	6.5960%	6.6730%	6.6458%	6.7359%
Staphylococcus	47.7054%	47.7567%	47.9460%	48.4683%	70.4825%	70.6257%	69.6443%	69.7634%
Stenotrophomonas	0.0000%	0.0000%	0.0000%	0.0000%	0.0054%	0.0054%	0.0055%	0.0056%
Streptococcus	5.1850%	5.6216%	6.5368%	5.5004%	9.6412%	9.0696%	10.0777%	9.4791%

Table SN4.4. Results on the Illumina mock communities

This table shows the results obtained after quality filtering the four Illumina mock communities with different filtering methods. For each method, the "NoSUB" table shows the results obtained directly after filtering, while the "SUB" table shows the results obtained after performing 100 independent random sub-samplings of the filtered reads. For each sample, the number of reads kept by the most stringent method was chosen as the sub-sampling size for the rest of the methods. Abbreviations: std, standard deviation; Coverage, Good's coverage; TaxBias, taxonomic bias (see methods).

Even1M

Poisson binomial filtering

NoSUB

OTUs	Singletons	Coverage	TaxBias	#Reads passing filters
41	17	0.9995	0.0490	35081

Perfect	Good	Noisy	Contaminant	Other
17	4	1	0	19

SUB_28517

OTUs	OTUs_std	Singletons	Singletons_std	Coverage	Coverage_std
38.14	1.84	15.7	2.18	0.9994	0.0001

Perfect	Good	Noisy	Contaminant	Other
17	3.67	1.53	0	15.94

mothur

NoSUB

OTUs	Singletons	Coverage	TaxBias	#Reads passing filters
66	41	0.9988	0.0426	34040

Perfect	Good	Noisy	Contaminant	Other
17	4	1	1	43

SUB_28517

OTUs	OTUs_std	Singletons	Singletons_std	Coverage	Coverage_std
59.45	2.46	35.56	2.41	0.9988	0.0001

Perfect	Good	Noisy	Contaminant	Other
17	3.51	1.76	0.87	36.31

QIIME

NoSUB

OTUs	Singletons	Coverage	CompBias	#Reads passing filters
112	77	0.9979	0.0337	36651

Perfect	Good	Noisy	Contaminant	Other
17	5	8	0	82

SUB_28517

OTUs	OTUs_std	Singletons	Singletons_std	Coverage	Coverage_std
93.77	3.94	64.2	3.98	0.9977	0.0001

Perfect	Good	Noisy	Contaminant	Other
17	4.41	6.35	0	66.01

USEARCH expected errors

NoSUB

OTUs	Singletons	Coverage	TaxBias	#Reads passing filters
61	32	0.9991	0.0461	34578

Perfect	Good	Noisy	Contaminant	Other
17	4	4	2	34

SUB_28517

OTUs	OTUs_std	Singletons	Singletons_std	Coverage	Coverage_std
55.19	2.49	28.98	2.69	0.9990	0.0001

Perfect	Good	Noisy	Contaminant	Other
17.11	3.82	3.99	1.63	28.64

Quality-trimming

NoSUB

OTUs	Singletons	Coverage	TaxBias	#Reads passing filters
44	21	0.9993	0.0678	28517

Perfect	Good	Noisy	Contaminant	Other
17	3.67	1.53	0	15.94

Even2M

Poisson binomial filtering

NoSUB

OTUs	Singletons	Coverage	TaxBias	#Reads passing filters
29	6	0.9998	0.0558	33841
**Perfect**	**Good**	**Noisy**	**Contaminant**	**Other**
18	3	0	0	8

SUB_26471

OTUs	OTUs_std	Singletons	Singletons_std	Coverage	Coverage_std
27.53	1.05	5.3	1.03	0.9998	0.0000
**Perfect**	**Good**	**Noisy**	**Contaminant**	**Other**	
17.96	2.69	0.32	0	6.56	

mothur

NoSUB

OTUs	Singletons	Coverage	TaxBias	#Reads passing filters
67	41	0.9988	0.0481	32849
**Perfect**	**Good**	**Noisy**	**Contaminant**	**Other**
18	3	4	0	42

SUB_26471

OTUs	OTUs_std	Singletons	Singletons_std	Coverage	Coverage_std
58.23	2.52	33.66	2.4	0.9987	0.0001
**Perfect**	**Good**	**Noisy**	**Contaminant**	**Other**	
18	2.78	2.85	0	34.6	

QIIME

NoSUB

OTUs	Singletons	Coverage	CompBias	#Reads passing filters
129	89	0.9975	0.0392	35741
**Perfect**	**Good**	**Noisy**	**Contaminant**	**Other**
18	4	14	2	91

SUB_26471

OTUs	OTUs_std	Singletons	Singletons_std	Coverage	Coverage_std
103.61	4.66	70.35	4.71	0.9973	0.0002
**Perfect**	**Good**	**Noisy**	**Contaminant**	**Other**	
18	3.89	9.58	1.39	70.75	

USEARCH expected errors

NoSUB

OTUs	Singletons	Coverage	TaxBias	#Reads passing filters
53	25	0.9992	0.0559	33130
**Perfect**	**Good**	**Noisy**	**Contaminant**	**Other**
18	4	3	0	28

SUB_26471

OTUs	OTUs_std	Singletons	Singletons_std	Coverage	Coverage_std
47.31	2.31	21.32	2.45	0.9992	0.0001
**Perfect**	**Good**	**Noisy**	**Contaminant**	**Other**	
18	3.83	2.59	0	22.89	

Quality-trimming

NoSUB

OTUs	Singletons	Coverage	TaxBias	#Reads passing filters
40	12	0.9995	0.0818	26471
**Perfect**	**Good**	**Noisy**	**Contaminant**	**Other**
18	4	1	0	17

Stag1M

Poisson binomial filtering

NoSUB

OTUs	Singletons	Coverage	TaxBias	#Reads passing filters
29	12	0.9996	0.0342	33497

Perfect	Good	Noisy	Contaminant	Other
16	2	0	3	8

SUB_26431

OTUs	OTUs_std	Singletons	Singletons_std	Coverage	Coverage_std
26.61	1.3	9.92	1.4	0.9996	0.0001

Perfect	Good	Noisy	Contaminant	Other
15.94	1.57	0.06	2.65	6.39

mothur

NoSUB

OTUs	Singletons	Coverage	TaxBias	#Reads passing filters
77	57	0.9982	0.0204	32406

Perfect	Good	Noisy	Contaminant	Other
16	1	4	4	52

SUB_26431

OTUs	OTUs_std	Singletons	Singletons_std	Coverage	Coverage_std
65.92	2.85	47.06	2.95	0.9982	0.0001

Perfect	Good	Noisy	Contaminant	Other
15.97	0.78	3.37	3.39	42.41

QIIME

NoSUB

OTUs	Singletons	Coverage	CompBias	#Reads passing filters
111	84	0.9976	0.0223	34751

Perfect	Good	Noisy	Contaminant	Other
16	2	6	4	83

SUB_26431

OTUs	OTUs_std	Singletons	Singletons_std	Coverage	Coverage_std
89.26	3.79	65.63	4.05	0.9975	0.0002

Perfect	Good	Noisy	Contaminant	Other
15.98	1.7	4.51	3.29	63.78

USEARCH expected errors

NoSUB

OTUs	Singletons	Coverage	TaxBias	#Reads passing filters
44	22	0.9993	0.0244	32671

Perfect	Good	Noisy	Contaminant	Other
16	1	6	3	18

SUB_26431

OTUs	OTUs_std	Singletons	Singletons_std	Coverage	Coverage_std
39.29	1.87	19.32	2.13	0.9993	0.0001

Perfect	Good	Noisy	Contaminant	Other
15.7	1	4.65	2.78	15.16

Quality-trimming

NoSUB

OTUs	Singletons	Coverage	TaxBias	#Reads passing filters
30	11	0.9996	0.1063	26431

Perfect	Good	Noisy	Contaminant	Other
15	0	2	3	10

Stag2M

Poisson binomial filtering

NoSUB

OTUs	Singletons	Coverage	TaxBias	#Reads passing filters
24	6	0.9998	0.0262	32465

Perfect	Good	Noisy	Contaminant	Other
17	1	1	1	4

SUB_26471

OTUs	OTUs_std	Singletons	Singletons_std	Coverage	Coverage_std
22.46	1.11	4.72	1.22	0.9998	0.0000

Perfect	Good	Noisy	Contaminant	Other
16.97	0.69	0.9	0.73	3.17

mothur

NoSUB

OTUs	Singletons	Coverage	TaxBias	#Reads passing filters
75	52	0.9983	0.0217	31319

Perfect	Good	Noisy	Contaminant	Other
17	1	4	1	52

SUB_26471

OTUs	OTUs_std	Singletons	Singletons_std	Coverage	Coverage_std
63.79	2.85	42.97	3.04	0.9983	0.0001

Perfect	Good	Noisy	Contaminant	Other
16.97	0.77	2.85	0.79	42.41

QIIME

NoSUB

OTUs	Singletons	Coverage	CompBias	#Reads passing filters
112	82	0.9976	0.0270	33758

Perfect	Good	Noisy	Contaminant	Other
16	1	13	1	81

SUB_26471

OTUs	OTUs_std	Singletons	Singletons_std	Coverage	Coverage_std
89.04	4.45	63.41	4.35	0.9975	0.0002

Perfect	Good	Noisy	Contaminant	Other
16.33	0.66	9.44	0.73	61.88

USEARCH expected errors

NoSUB

OTUs	Singletons	Coverage	TaxBias	#Reads passing filters
42	20	0.9994	0.0391	31454

Perfect	Good	Noisy	Contaminant	Other
16	2	4	1	19

SUB_26471

OTUs	OTUs_std	Singletons	Singletons_std	Coverage	Coverage_std
37.34	2.15	16.95	2.28	0.9993	0.0001

Perfect	Good	Noisy	Contaminant	Other
15.96	1.68	3.21	0.85	15.64

Quality-trimming

NoSUB

OTUs	Singletons	Coverage	TaxBias	#Reads passing filters
28	10	0.9996	0.0984	26471

Perfect	Good	Noisy	Contaminant	Other
15	3	1	1	8

Table SN4.5. Taxonomic composition of the IonTorrent 16S mock community datasets

This table gives the taxonomic composition of the raw datasets from the 16S mock communities sequenced with the IonTorrent PGM platform, classified at the genus level with mothur's *classify.seqs* command.

	Even1P	Even2P
Propionibacterium	5.9725%	0.0359%
Enterococcus	2.5484%	3.1057%
Lactobacillus	4.4973%	5.2174%
Pseudomonas	0.5743%	1.5448%
Listeria	6.6078%	6.1435%
Actinobacillus	0.0108%	0.0000%
Bacillus	3.3918%	3.6685%
Rhodobacter	2.0997%	2.6226%
Enterobacter	0.0467%	0.0399%
Salmonella	0.0036%	0.0000%
Escherichia_Shigella	2.4120%	1.7484%
Helicobacter	14.3929%	14.1232%
Actinomyces	3.2375%	0.0000%
Neisseria	8.7003%	7.7043%
Citrobacter	0.0000%	0.0160%
Clostridium_sensu_stricto	4.1671%	5.3411%
Bacteroides	11.8660%	15.4844%
Streptococcus	12.6736%	14.4425%
Yokenella	0.0036%	0.0120%
Staphylococcus	7.9753%	9.6363%
Acinetobacter	3.8046%	5.4529%
Deinococcus	5.0142%	3.6605%

Table SN4.6. Results on the IonTorrent mock communities

This table shows the results obtained after quality filtering the two IonTorrent mock communities with different filtering methods. For each method, the “NoSUB” table shows the results obtained directly after filtering, while the “SUB” table shows the results obtained after performing 100 independent random sub-samplings of the filtered reads. For each sample, the number of reads kept by the most stringent method was chosen as the sub-sampling size for the rest of the methods. Abbreviations: std, standard deviation; Coverage, Good's coverage; TaxBias, taxonomic bias (see methods).

Even1P

Poisson binomial filtering

NoSUB

OTUs	Singletons	Coverage	CompBias	#Reads passing filters
24	4	0.9997	0.1642	12303

Perfect	Good	Noisy	Contaminant	Other
16	3	3	0	2

SUB_6850

OTUs	OTUs_std	Singletons	Singletons_std	Coverage	Coverage_std
21.79	1.11	3.22	1.22	0.9995	0.0002

Perfect	Good	Noisy	Contaminant	Other
15.55	2.83	2.33	0	1.08

USEARCH expected errors

NoSUB

OTUs	Singletons	Coverage	CompBias	#Reads passing filters
38	15	0.9978	0.2223	6850

Perfect	Good	Noisy	Contaminant	Other
16	4	6	0	12

Even2P

Poisson binomial filtering

NoSUB

OTUs	Singletons	Coverage	CompBias	#Reads passing filters
30	9	0.9993	0.1159	12644

Perfect	Good	Noisy	Contaminant	Other
12	4	4	0	10

SUB_7432

OTUs	OTUs_std	Singletons	Singletons_std	Coverage	Coverage_std
26.45	1.44	5.72	1.52	0.9992	0.0002

Perfect	Good	Noisy	Contaminant	Other
11.92	4.25	2.93	0.08	7.27

USEARCH expected errors

NoSUB

OTUs	Singletons	Coverage	CompBias	#Reads passing filters
43	16	0.9978	0.1454	7432

Perfect	Good	Noisy	Contaminant	Other
12	5	4	1	21

Supplementary Note 5. Results on environmental communities

SN5.1. Performance of the different filtering methods on the environmental communities datasets

The performance of the different filtering methods was also evaluated by quality-filtering publicly available datasets obtained by sequencing environmental communities (**Supplementary Figures SN5.1, SN5.2, SN5.3**) The results were similar to the ones obtained with the mock communities, with Poisson binomial filtering filtering being the most consistent method in producing the smallest number of OTUs and singletons. Additionally, the OTUs obtained with PBF were overall the most similar to the 16S sequences present in the SILVA 16S reference alignment (Quast *et al.,* 2013), which suggests that they contained the smallest number of errors. In the environmental 454 datasets, PyroNoise showed better results than in the 454 mock communities, but did it in an irregular fashion, especially in terms of OTU accuracy (**Supplementary Figure SN5.1d**). This inconsistency may be again due to the alteration of the original reads, and suggests that PyroNoise requires a finer parameter optimization than other approaches in order to be fully effective. In the environmental IonTorrent datasets PyroNoise discarded the smallest number of reads, but resulted in the highest number of singletons and OTUs, which also beared the least similarity to the reference alignment. USEARCH showed an intermediate performance between PyroNoise and Poisson binomial filtering (**Supplementary Figure SN5.3)**. Finally, in the environmental Illumina datasets all filtering methods showed a similar behaviour to that in the mock communities (**Supplementary Figure SN5.2**).

Figure SN5.1. Results on 454 environmental communities

(a, b): Number of singletons (a, bars), total species (a, symbols) and reads (c) retrieved after filtering the raw reads with the different methods and performing chimera removal and clustering with a common pipeline. OTU and singleton numbers were obtained by averaging the results from 100 independent library size standardizations.
(c): Taxonomic bias caused by the different filtering methods, measured as the Bray-Curtis dissimilarity between the raw and the filtered read communities.
(d): Average percent OTU similarity to their best hit from the SILVA bacterial 16S reference alignment. This number was obtained by averaging the results from 100 independent library size standardizations.

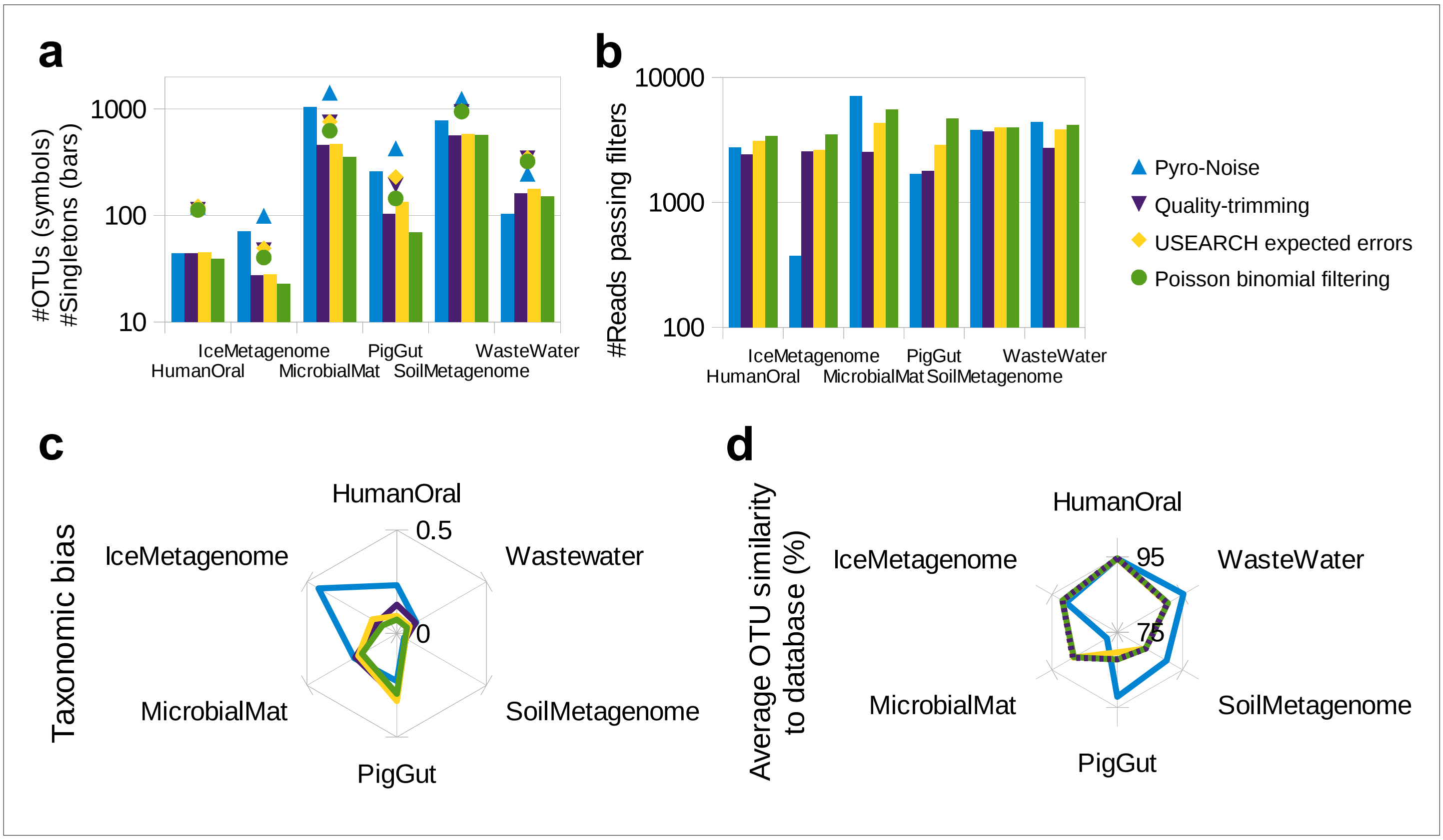

Table SN5.1. Results on the 454 environmental communities

This table shows the results obtained after quality filtering the six 454 environmental communities with different filtering methods. For each method, the “NoSUB” table shows the results obtained directly after filtering, while the “SUB” table shows the results obtained after performing 100 independent random sub-samplings of the filtered reads. For each sample, the number of reads kept by the most stringent method was chosen as the sub-sampling size for the rest of the methods. Abbreviations: std, standard deviation; Coverage, Good's coverage; TaxBias, taxonomic bias (see methods); OTUsim, average OTU similarity to their best hit from the SILVA bacterial 16S reference alignment.

HumanOral

Poisson binomial filtering

NoSUB

OTUs	Singletons	Coverage	OTUsim	OTUsim_std	TaxBias	#Reads passing filters
127	43	0.9874	94.61	3.42	0.0672	3409

SUB_2418

OTUs	OTUs_std	Singletons	Singletons_std	Coverage	Coverage_std	OTUsim	OTUsim_std
112.66	2.38	39.02	2.77	0.9839	0.0011	94.69	3.4

PyroNoise

NoSUB

OTUs	Singletons	Coverage	OTUsim	OTUsim_std	TaxBias	#Reads passing filters
126	47	0.9830	94.52	6.57	0.2330	2758

SUB_2418

OTUs	OTUs_std	Singletons	Singletons_std	Coverage	Coverage_std	OTUsim	OTUsim_std
120.6	1.68	44.07	1.92	0.9818	0.0008	94.75	6.47

USEARCH expected errors

NoSUB

OTUs	Singletons	Coverage	OTUsim	OTUsim_std	TaxBias	#Reads passing filters
133	47	0.9849	94.54	3.41	0.0858	3110

SUB_2418

OTUs	OTUs_std	Singletons	Singletons_std	Coverage	Coverage_std	OTUsim	OTUsim_std
122.03	1.92	45.1	2.89	0.9813	0.0012	94.66	3.44

Quality-trimming

NoSUB

OTUs	Singletons	Coverage	OTUsim	OTUsim_std	TaxBias	#Reads passing filters
113	44	0.9818	94.69	3.39	0.1391	2418

IceMetagenome

Poisson binomial filtering

NoSUB

OTUs	Singletons	Coverage	OTUsim	OTUsim_std	TaxBias	#Reads passing filters
127	60	0.9830	90.97	6.63	0.1361	3519

SUB_375

OTUs	OTUs_std	Singletons	Singletons_std	Coverage	Coverage_std	OTUsim	OTUsim_std
40.26	4.27	22.8	4.24	0.9390	0.0113	91.8	6.13

PyroNoise

NoSUB

OTUs	Singletons	Coverage	OTUsim	OTUsim_std	TaxBias	#Reads passing filters
99	71	0.8107	90.72	4.87	0.4366	375

USEARCH expected errors

NoSUB

OTUs	Singletons	Coverage	OTUsim	OTUsim_std	TaxBias	#Reads passing filters
134	63	0.9762	90.6	7.01	0.0796	2644

SUB_375

OTUs	OTUs_std	Singletons	Singletons_std	Coverage	Coverage_std	OTUsim	OTUsim_std
49.18	4.46	27.99	4.54	0.9254	0.0121	91.77	6.36

Quality-trimming

NoSUB

OTUs	Singletons	Coverage	OTUsim	OTUsim_std	TaxBias	#Reads passing filters
133	60	0.9765	91.2	6.24	0.1086	2555

SUB_375

OTUs	OTUs_std	Singletons	Singletons_std	Coverage	Coverage_std	OTUsim	OTUsim_std
47.13	4.43	27.54	4.79	0.9266	0.0128	91.78	6.22

MicrobialMat

Poisson binomial filtering

NoSUB

OTUs	Singletons	Coverage	OTUsim	OTUsim_std	TaxBias	#Reads passing filters
961	494	0.9110	88.02	7.02	0.1948	5549

SUB_2543

OTUs	OTUs_std	Singletons	Singletons_std	Coverage	Coverage_std	OTUsim	OTUsim_std
624.59	13.11	353.49	14.49	0.8610	0.0057	88.3	6.97

PyroNoise

NoSUB

OTUs	Singletons	Coverage	OTUsim	OTUsim_std	TaxBias	#Reads passing filters
2973	2042	0.7140	77.86	9.6	0.2380	7140

SUB_2543

OTUs	OTUs_std	Singletons	Singletons_std	Coverage	Coverage_std	OTUsim	OTUsim_std
1418.56	18.86	1048.85	24.58	0.5876	0.0097	78.18	9.64

USEARCH expected errors

NoSUB

OTUs	Singletons	Coverage	OTUsim	OTUsim_std	TaxBias	#Reads passing filters
1058	616	0.8584	88.32	6.82	0.2146	4350

SUB_2543

OTUs	OTUs_std	Singletons	Singletons_std	Coverage	Coverage_std	OTUsim	OTUsim_std
761.12	11.96	467.58	13.72	0.8161	0.0054	88.53	6.76

Quality-trimming

NoSUB

OTUs	Singletons	Coverage	OTUsim	OTUsim_std	TaxBias	#Reads passing filters
751	460	0.8191	88.61	6.45	0.2311	2543

PigGut

Poisson binomial filtering

NoSUB

OTUs	Singletons	Coverage	OTUsim	OTUsim_std	TaxBias	#Reads passing filters
232	103	0.9781	82.04	8.86	0.2910	4707

SUB_1701

OTUs	OTUs_std	Singletons	Singletons_std	Coverage	Coverage_std	OTUsim	OTUsim_std
144.5	5.97	69.06	7.14	0.9594	0.0042	82.23	8.76

PyroNoise

NoSUB

OTUs	Singletons	Coverage	OTUsim	OTUsim_std	TaxBias	#Reads passing filters
426	258	0.8483	92.15	4.55	0.2303	1701

USEARCH expected errors

NoSUB

OTUs	Singletons	Coverage	OTUsim	OTUsim_std	TaxBias	#Reads passing filters
313	184	0.9366	80.31	8.65	0.3267	2900

SUB_1701

OTUs	OTUs_std	Singletons	Singletons_std	Coverage	Coverage_std	OTUsim	OTUsim_std
229.79	5.73	133.8	7.11	0.9213	0.0042	80.34	8.62

Quality-trimming

NoSUB

OTUs	Singletons	Coverage	OTUsim	OTUsim_std	TaxBias	#Reads passing filters
199	106	0.9405	82.17	9.05	0.3082	1782

SUB_1701

OTUs	OTUs_std	Singletons	Singletons_std	Coverage	Coverage_std	OTUsim	OTUsim_std
194.14	1.95	103.52	2.53	0.9391	0.0015	82.2	9.04

SoilMetagenome

Poisson binomial filtering

NoSUB

OTUs	Singletons	Coverage	OTUsim	OTUsim_std	TaxBias	#Reads passing filters
990	588	0.8528	83.67	5.04	0.0472	3995

SUB_3712

OTUs	OTUs_std	Singletons	Singletons_std	Coverage	Coverage_std	OTUsim	OTUsim_std
946.79	6.25	569.97	7.07	0.8465	0.0019	83.7	5.04

PyroNoise

NoSUB

OTUs	Singletons	Coverage	OTUsim	OTUsim_std	TaxBias	#Reads passing filters
1266	796	0.7912	90.07	5.22	0.0406	3813

SUB_3712

OTUs	OTUs_std	Singletons	Singletons_std	Coverage	Coverage_std	OTUsim	OTUsim_std
1244.62	5.07	782.37	5.74	0.7892	0.0015	90.09	5.21

USEARCH expected errors

NoSUB

OTUs	Singletons	Coverage	OTUsim	OTUsim_std	TaxBias	#Reads passing filters
1003	597	0.8507	83.67	5.13	0.0495	3999

SUB_3712

OTUs	OTUs_std	Singletons	Singletons_std	Coverage	Coverage_std	OTUsim	OTUsim_std
958.18	6.07	578.84	8.25	0.8441	0.0022	83.71	5.12

Quality-trimming

NoSUB

OTUs	Singletons	Coverage	OTUsim	OTUsim_std	TaxBias	#Reads passing filters
941	565	0.8478	83.75	4.95	0.0494	3712

WasteWater

Poisson binomial filtering

NoSUB

OTUs	Singletons	Coverage	OTUsim	OTUsim_std	TaxBias	#Reads passing filters
390	169	0.9597	90.07	8.4	0.0567	4191

SUB_2731

OTUs	OTUs_std	Singletons	Singletons_std	Coverage	Coverage_std	OTUsim	OTUsim_std
321.81	7	151.1	8.66	0.9447	0.0032	90.5	8.23

PyroNoise

NoSUB

OTUs	Singletons	Coverage	OTUsim	OTUsim_std	TaxBias	#Reads passing filters
297	109	0.9752	95.11	4.18	0.1083	4397

SUB_2731

OTUs	OTUs_std	Singletons	Singletons_std	Coverage	Coverage_std	OTUsim	OTUsim_std
245.22	5.73	102.81	5.54	0.9624	0.0020	95.27	4.15

USEARCH expected errors

NoSUB

OTUs	Singletons	Coverage	OTUsim	OTUsim_std	TaxBias	#Reads passing filters
412	197	0.9489	89.88	8.47	0.0718	3853

SUB_2731

OTUs	OTUs_std	Singletons	Singletons_std	Coverage	Coverage_std	OTUsim	OTUsim_std
348.56	6.41	176.9	6.79	0.9352	0.0025	90.26	8.31

Quality-trimming

NoSUB

OTUs	Singletons	Coverage	OTUsim	OTUsim_std	TaxBias	#Reads passing filters
345	160	0.9414	90.33	8.29	0.1050	2731

Figure SN5.2. Results on the Illumina environmental communities

(a, b): Number of singletons (a, bars), total species (a, symbols) and reads (c) retrieved after filtering the raw reads with the different methods and performing chimera removal and clustering with a common pipeline. OTU and singleton numbers were obtained by averaging the results from 100 independent library size standardizations.
(c): Taxonomic bias caused by the different filtering methods, measured as the Bray-Curtis dissimilarity between the raw and the filtered read communities.
(d): Average percent OTU similarity to their best hit from the SILVA bacterial 16S reference alignment. This number was obtained by averaging the results from 100 independent library size standardizations.

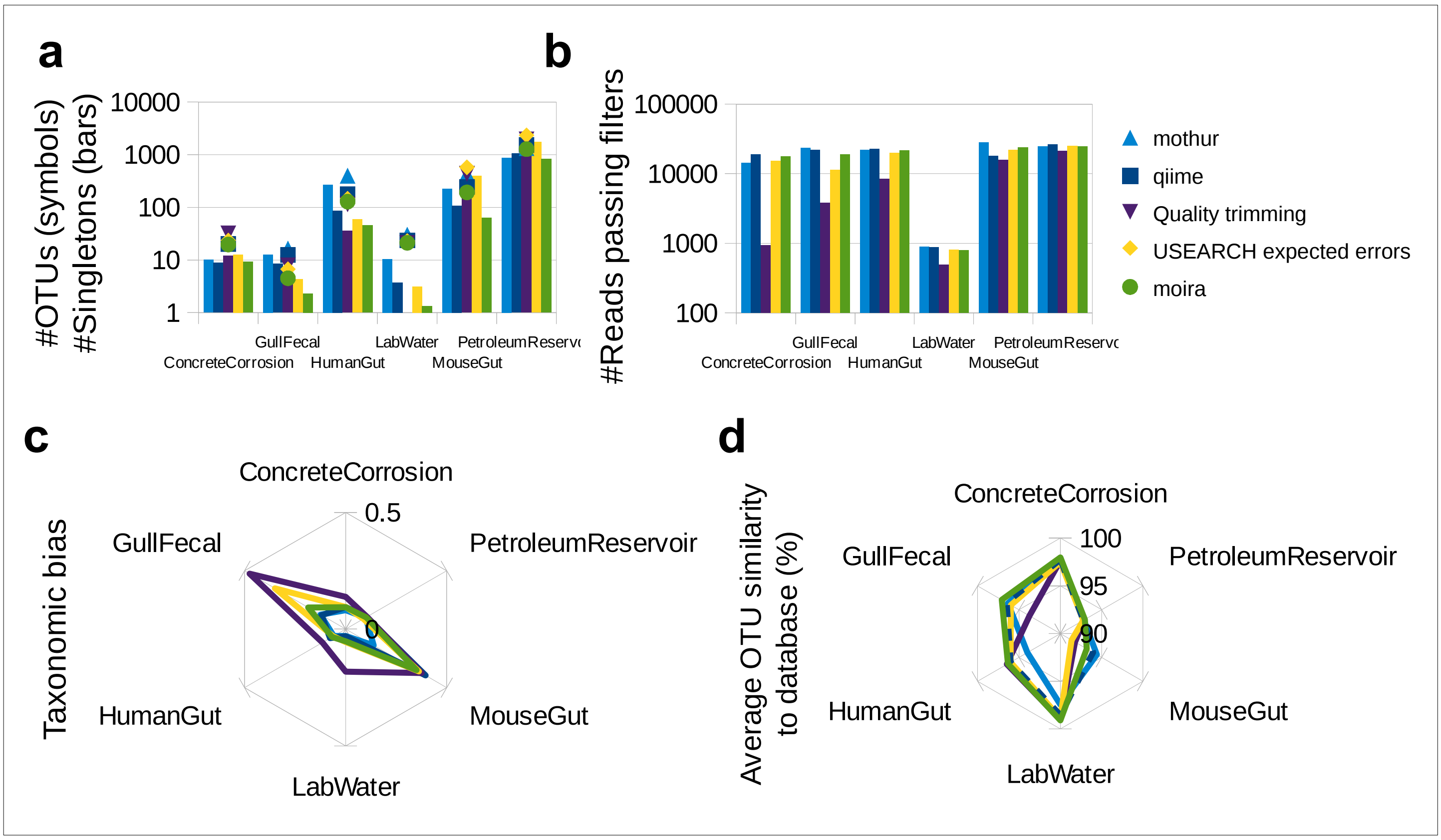

Table SN5.2. Results on the Illumina environmental communities

This table shows the results obtained after quality filtering the six Illumina environmental communities with different filtering methods. For each method, the “NoSUB” table shows the results obtained directly after filtering, while the “SUB” table shows the results obtained after performing 100 independent random sub-samplings of the filtered reads. For each sample, the number of reads kept by the most stringent method was chosen as the sub-sampling size for the rest of the methods. Abbreviations: std, standard deviation; Coverage, Good's coverage; TaxBias, taxonomic bias (see methods); OTUsim, average OTU similarity to their best hit from the SILVA bacterial 16S reference alignment.

ConcreteCorrosion

Poisson binomial filtering

NoSUB

OTUs	Singletons	Coverage	OTUsim	OTUsim_std	TaxBias	#Reads passing filters
78	34	0.9981	96.79	2.66	0.0940	17739

SUB_941

OTUs	OTUs_std	Singletons	Singletons_std	Coverage	Coverage_std	OTUsim	OTUsim_std
19.7	3.03	9.32	2.86	0.9901	0.0030	97.98	2.48

mothur

NoSUB

OTUs	Singletons	Coverage	OTUsim	OTUsim_std	TaxBias	#Reads passing filters
80	35	0.9976	96.57	3.33	0.0809	14379

SUB_941

OTUs	OTUs_std	Singletons	Singletons_std	Coverage	Coverage_std	OTUsim	OTUsim_std
20.63	2.8	10.1	2.69	0.9893	0.0029	97.94	2.67

QIIME

NoSUB

OTUs	Singletons	Coverage	OTUsim	OTUsim_std	CompBias	#Reads passing filters
101	56	0.9970	95.94	2.99	0.0951	18887

SUB_941

OTUs	OTUs_std	Singletons	Singletons_std	Coverage	Coverage_std	OTUsim	OTUsim_std
20.12	2.81	9.89	2.75	0.9895	0.0029	97.75	2.75

USEARCH expected errors

NoSUB

OTUs	Singletons	Coverage	OTUsim	OTUsim_std	TaxBias	#Reads passing filters
97	47	0.9969	96.1	2.86	0.0952	15247

SUB_941

OTUs	OTUs_std	Singletons	Singletons_std	Coverage	Coverage_std	OTUsim	OTUsim_std
23.55	2.86	12.63	2.81	0.9866	0.0030	97.51	2.64

Quality-trimming

NoSUB

OTUs	Singletons	Coverage	OTUsim	OTUsim_std	TaxBias	#Reads passing filters
32	12	0.9872	97.66	3.81	0.1385	941

GullFecal

Poisson binomial filtering

NoSUB

OTUs	Singletons	Coverage	OTUsim	OTUsim_std	TaxBias	#Reads passing filters
12	8	0.9996	95.26	5.14	0.1859	18905

SUB_3860

OTUs	OTUs_std	Singletons	Singletons_std	Coverage	Coverage_std	OTUsim	OTUsim_std
4.5	1.25	2.31	1.22	0.9994	0.0003	97.07	4.19

mothur

NoSUB

OTUs	Singletons	Coverage	OTUsim	OTUsim_std	TaxBias	#Reads passing filters
74	56	0.9976	95.55	2.8	0.1241	23628

SUB_3860

OTUs	OTUs_std	Singletons	Singletons_std	Coverage	Coverage_std	OTUsim	OTUsim_std
16.41	3.48	12.75	3.54	0.9967	0.0009	96.34	2.78

QIIME

NoSUB

OTUs	Singletons	Coverage	OTUsim	OTUsim_std	CompBias	#Reads passing filters
52	46	0.9979	95.45	3.32	0.1198	22223

SUB_3860

OTUs	OTUs_std	Singletons	Singletons_std	Coverage	Coverage_std	OTUsim	OTUsim_std
12.52	2.82	9.46	2.84	0.9975	0.0007	96.5	3.37

USEARCH expected errors

NoSUB

OTUs	Singletons	Coverage	OTUsim	OTUsim_std	TaxBias	#Reads passing filters
14	10	0.9991	94.94	4.79	0.3496	11344

SUB_3860

OTUs	OTUs_std	Singletons	Singletons_std	Coverage	Coverage_std	OTUsim	OTUsim_std
6.69	1.72	4.28	1.73	0.9989	0.0004	95.99	4.47

Quality-trimming

NoSUB

OTUs	Singletons	Coverage	OTUsim	OTUsim_std	TaxBias	#Reads passing filters
8	6	0.9984	93.74	5.64	0.4755	3860

HumanGut

Poisson binomial filtering

NoSUB

OTUs	Singletons	Coverage	OTUsim	OTUsim_std	TaxBias	#Reads passing filters
182	64	0.9970	95.94	3.67	0.0670	21675

SUB_8450

OTUs	OTUs_std	Singletons	Singletons_std	Coverage	Coverage_std	OTUsim	OTUsim_std
128.57	5.32	45.93	5.35	0.9946	0.0006	96.34	3.54

mothur

NoSUB

OTUs	Singletons	Coverage	OTUsim	OTUsim_std	TaxBias	#Reads passing filters
808	573	0.9742	93.43	4.09	0.0584	22218

SUB_8450

OTUs	OTUs_std	Singletons	Singletons_std	Coverage	Coverage_std	OTUsim	OTUsim_std
395.32	13.57	267.85	13.3	0.9683	0.0016	93.98	4.14

QIIME

NoSUB

OTUs	Singletons	Coverage	OTUsim	OTUsim_std	CompBias	#Reads passing filters
300	158	0.9930	95.53	3.4	0.0772	22556

SUB_8450

OTUs	OTUs_std	Singletons	Singletons_std	Coverage	Coverage_std	OTUsim	OTUsim_std
179.64	6.33	86.91	6.32	0.9897	0.0007	96.02	3.44

USEARCH expected errors

NoSUB

OTUs	Singletons	Coverage	OTUsim	OTUsim_std	TaxBias	#Reads passing filters
212	93	0.9953	95.69	3.61	0.0611	19811

SUB_8450

OTUs	OTUs_std	Singletons	Singletons_std	Coverage	Coverage_std	OTUsim	OTUsim_std
146.58	5.78	59.33	5.88	0.9930	0.0007	96.01	3.62

Quality-trimming

NoSUB

OTUs	Singletons	Coverage	OTUsim	OTUsim_std	TaxBias	#Reads passing filters
116	36	0.9957	96.45	3.4	0.1144	8450

LabWater

Poisson binomial filtering

NoSUB

OTUs	Singletons	Coverage	OTUsim	OTUsim_std	TaxBias	#Reads passing filters
22	2	0.9975	99.14	1.75	0.0518	799

SUB_491

OTUs	OTUs_std	Singletons	Singletons_std	Coverage	Coverage_std	OTUsim	OTUsim_std
21.22	0.73	1.34	0.75	0.9973	0.0015	99.1	1.77

mothur

NoSUB

OTUs	Singletons	Coverage	OTUsim	OTUsim_std	TaxBias	#Reads passing filters
39	19	0.9786	96.74	5	0.0294	888

SUB_491

OTUs	OTUs_std	Singletons	Singletons_std	Coverage	Coverage_std	OTUsim	OTUsim_std
30.1	2.12	10.43	2.22	0.9788	0.0045	97.46	4.4

QIIME

NoSUB

OTUs	Singletons	Coverage	OTUsim	OTUsim_std	CompBias	#Reads passing filters
29	9	0.9898	98.26	2.5	0.0306	879

SUB_491

OTUs	OTUs_std	Singletons	Singletons_std	Coverage	Coverage_std	OTUsim	OTUsim_std
24.58	1.56	4.74	1.59	0.9903	0.0032	98.54	2.35

USEARCH expected errors

NoSUB

OTUs	Singletons	Coverage	OTUsim	OTUsim_std	TaxBias	#Reads passing filters
25	5	0.9938	98.76	1.97	0.0559	808

SUB_491

OTUs	OTUs_std	Singletons	Singletons_std	Coverage	Coverage_std	OTUsim	OTUsim_std
22.91	1.1	3.14	1.23	0.9936	0.0025	98.85	1.93

Quality-trimming

NoSUB

OTUs	Singletons	Coverage	OTUsim	OTUsim_std	TaxBias	#Reads passing filters
21	1	0.9980	99.02	1.77	0.1823	491

MouseGut

Poisson binomial filtering

NoSUB

OTUs	Singletons	Coverage	OTUsim	OTUsim_std	TaxBias	#Reads passing filters
222	80	0.9966	93.1	3.46	0.3498	23805

SUB_15794

OTUs	OTUs_std	Singletons	Singletons_std	Coverage	Coverage_std	OTUsim	OTUsim_std
192.5	4.73	63.23	4.77	0.9960	0.0003	93.19	3.42

mothur

NoSUB

OTUs	Singletons	Coverage	OTUsim	OTUsim_std	TaxBias	#Reads passing filters
614	341	0.9880	94.44	3.27	0.1391	28341

SUB_15794

OTUs	OTUs_std	Singletons	Singletons_std	Coverage	Coverage_std	OTUsim	OTUsim_std
428.74	11.02	226.25	11.38	0.9857	0.0007	94.43	3.29

QIIME

NoSUB

OTUs	Singletons	Coverage	OTUsim	OTUsim_std	CompBias	#Reads passing filters
262	118	0.9934	93.97	3.08	0.3949	17994

SUB_15794

OTUs	OTUs_std	Singletons	Singletons_std	Coverage	Coverage_std	OTUsim	OTUsim_std
245.32	4.17	108.39	4.74	0.9931	0.0003	93.96	3.09

USEARCH expected errors

NoSUB

OTUs	Singletons	Coverage	OTUsim	OTUsim_std	TaxBias	#Reads passing filters
732	522	0.9763	91.19	3.75	0.3619	22067

SUB_15794

OTUs	OTUs_std	Singletons	Singletons_std	Coverage	Coverage_std	OTUsim	OTUsim_std
575.78	10.28	399.21	10.27	0.9747	0.0007	91.38	3.77

Quality-trimming

NoSUB

OTUs	Singletons	Coverage	OTUsim	OTUsim_std	TaxBias	#Reads passing filters
436	262	0.9834	91.79	3.62	0.3750	15794

PetroleumReservoir

Poisson binomial filtering

NoSUB

OTUs	Singletons	Coverage	OTUsim	OTUsim_std	TaxBias	#Reads passing filters
1416	921	0.9630	92.9	5.18	0.1015	24917

SUB_21410

OTUs	OTUs_std	Singletons	Singletons_std	Coverage	Coverage_std	OTUsim	OTUsim_std
1279.65	11.6	831.23	13.12	0.9612	0.0006	92.97	5.21

mothur

NoSUB

OTUs	Singletons	Coverage	OTUsim	OTUsim_std	TaxBias	#Reads passing filters
1466	976	0.9603	92.78	5.42	0.0983	24593

SUB_21410

OTUs	OTUs_std	Singletons	Singletons_std	Coverage	Coverage_std	OTUsim	OTUsim_std
1332.13	11.63	882.88	11.9	0.9588	0.0006	92.86	5.41

QIIME

NoSUB

OTUs	Singletons	Coverage	OTUsim	OTUsim_std	CompBias	#Reads passing filters
1775	1241	0.9531	92.71	5.41	0.1038	26485

SUB_21410

OTUs	OTUs_std	Singletons	Singletons_std	Coverage	Coverage_std	OTUsim	OTUsim_std
1525.32	12.96	1060.31	13.92	0.9505	0.0007	92.8	5.47

USEARCH expected errors

NoSUB

OTUs	Singletons	Coverage	OTUsim	OTUsim_std	TaxBias	#Reads passing filters
2669	1986	0.9218	92.73	5.14	0.0880	25385

SUB_21410

OTUs	OTUs_std	Singletons	Singletons_std	Coverage	Coverage_std	OTUsim	OTUsim_std
2339.45	17.61	1743.07	19.69	0.9186	0.0009	92.78	5.16

Quality-trimming

NoSUB

OTUs	Singletons	Coverage	OTUsim	OTUsim_std	TaxBias	#Reads passing filters
1963	1397	0.9348	92.87	5.18	0.1057	21410

Figure SN5.3. Results on the IonTorrent environmental communities

(a, b): Number of singletons (a, bars), total species (a, symbols) and reads (c) retrieved after filtering the raw reads with the different methods and performing chimera removal and clustering with a common pipeline. OTU and singleton numbers were obtained by averaging the results from 100 independent library size standardizations.
(c): Taxonomic bias caused by the different filtering methods, measured as the Bray-Curtis dissimilarity between the raw and the filtered read communities.
(d): Average percent OTU similarity to their best hit from the SILVA bacterial 16S reference alignment. This number was obtained by averaging the results from 100 independent library size standardizations.

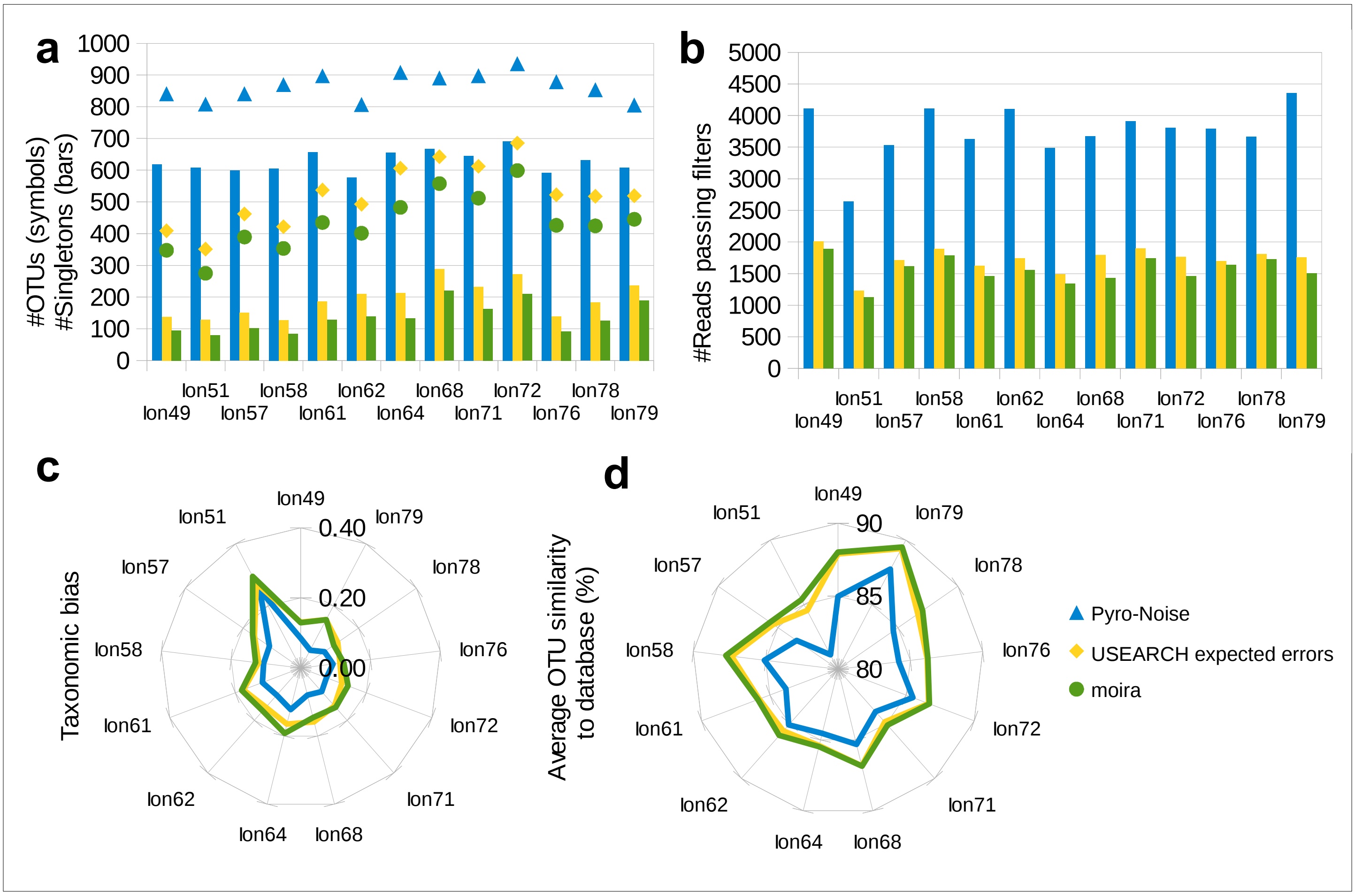

Table SN5.3. Results on the IonTorrent environmental communities

This table shows the results obtained after quality filtering the thirteen IonTorrent environmental communities with different filtering methods. For each method, the “NoSUB” table shows the results obtained directly after filtering, while the “SUB” table shows the results obtained after performing 100 independent random sub-samplings of the filtered reads. The number of reads kept in sample Ion51 by Poisson binomial filtering was used as the subsampling size for all the datasets. Abbreviations: std, standard deviation; Coverage, Good's coverage; TaxBias, taxonomic bias (see methods); OTUsim, average OTU similarity to their best hit from the SILVA bacterial 16S reference alignment.

Ion49

Poisson binomial filtering

NoSUB

OTUs	Singletons	Coverage	OTUsim	OTUsim_std	CompBias	#Reads passing filters
462	112	0.44	87.81	8.3	0.1290	1895

SUB_1124

OTUs	OTUs_std	Singletons	Singletons_std	Coverage	Coverage_std	OTUsim	OTUsim_std
347.49	7.33	95	5.98	0.4097	0.4098	88.03	8.2

PyroNoise

NoSUB

OTUs	Singletons	Coverage	OTUsim	OTUsim_std	CompBias	#Reads passing filters
2481	1651	0.2608	84.54	7.6	0.0850	4108

SUB_1124

OTUs	OTUs_std	Singletons	Singletons_std	Coverage	Coverage_std	OTUsim	OTUsim_std
840.41	12.46	618.05	14.86	0.1741	0.1744	84.98	7.55

USEARCH expected errors

NoSUB

OTUs	Singletons	Coverage	OTUsim	OTUsim_std	CompBias	#Reads passing filters
582	184	0.4171	87.56	8.26	0.1056	2014

SUB_1124

OTUs	OTUs_std	Singletons	Singletons_std	Coverage	Coverage_std	OTUsim	OTUsim_std
409.02	8.76	137.63	8.42	0.3844	0.3845	87.91	8.09

Ion51

Poisson binomial filtering

NoSUB

OTUs	Singletons	Coverage	OTUsim	OTUsim_std	CompBias	#Reads passing filters
275	80	0.4302	85.39	10.75	0.2951	1124

PyroNoise

NoSUB

OTUs	Singletons	Coverage	OTUsim	OTUsim_std	CompBias	#Reads passing filters
1651	1126	0.2477	80.68	10.35	0.1290	2640

SUB_1124

OTUs	OTUs_std	Singletons	Singletons_std	Coverage	Coverage_std	OTUsim	OTUsim_std
807.82	11.55	607.53	14.9	0.1906	0.2506	81.11	10.37

USEARCH expected errors

NoSUB

OTUs	Singletons	Coverage	OTUsim	OTUsim_std	CompBias	#Reads passing filters
373	129	0.4050	84.5	10.73	0.2726	1232

SUB_1124

OTUs	OTUs_std	Singletons	Singletons_std	Coverage	Coverage_std	OTUsim	OTUsim_std
351.16	4.15	128.87	4.84	0.4013	0.4013	84.55	10.7

Ion57

Poisson binomial filtering

NoSUB

OTUs	Singletons	Coverage	OTUsim	OTUsim_std	CompBias	#Reads passing filters
480	115	0.4158	85.44	10.07	0.1670	1615

SUB_1124

OTUs	OTUs_std	Singletons	Singletons_std	Coverage	Coverage_std	OTUsim	OTUsim_std
389.31	7.43	101.75	6.79	0.3949	0.3949	85.66	9.96

PyroNoise

NoSUB

OTUs	Singletons	Coverage	OTUsim	OTUsim_std	CompBias	#Reads passing filters
2208	1429	0.2496	82.94	8.92	0.1091	3532

SUB_1124

OTUs	OTUs_std	Singletons	Singletons_std	Coverage	Coverage_std	OTUsim	OTUsim_std
840.49	11.44	599.03	14.22	0.1742	0.1745	83.44	8.91

USEARCH expected errors

NoSUB

OTUs	Singletons	Coverage	OTUsim	OTUsim_std	CompBias	#Reads passing filters
604	180	0.3905	85.13	10.27	0.1605	1712

SUB_1124

OTUs	OTUs_std	Singletons	Singletons_std	Coverage	Coverage_std	OTUsim	OTUsim_std
461.75	7.86	150.49	8.61	0.3644	0.3645	85.4	10.13

Ion58

Poisson binomial filtering

**NoSUB**							
**OTUs**	**Singletons**	**Coverage**	**OTUsim**	**OTUsim_std**	**CompBias**	**#Reads passing filters**	
452	98	0.4335	87.45	8.88	0.1298	1783	
**SUB_1124**							
**OTUs**	**OTUs_std**	**Singletons**	**Singletons_std**	**Coverage**	**Coverage_std**	**OTUsim**	**OTUsim_std**
353.35	7.37	84.49	6.15	0.4131	0.4131	87.74	8.68

PyroNoise

**NoSUB**							
**OTUs**	**Singletons**	**Coverage**	**OTUsim**	**OTUsim_std**	**CompBias**	**#Reads passing filters**	
2538	1609	0.2590	84.58	7.52	0.1069	4108	
**SUB_1124**							
**OTUs**	**OTUs_std**	**Singletons**	**Singletons_std**	**Coverage**	**Coverage_std**	**OTUsim**	**OTUsim_std**
869.3	11.53	605.04	14.33	0.1685	0.1687	85.09	7.4

USEARCH expected errors

**NoSUB**							
**OTUs**	**Singletons**	**Coverage**	**OTUsim**	**OTUsim_std**	**CompBias**	**#Reads passing filters**	
576	164	0.4084	86.9	9.12	0.1211	1888	
**SUB_1124**							
**OTUs**	**OTUs_std**	**Singletons**	**Singletons_std**	**Coverage**	**Coverage_std**	**OTUsim**	**OTUsim_std**
421.76	9.22	127.94	7.87	0.3838	0.3838	87.35	8.86

Ion61

Poisson binomial filtering

**NoSUB**							
**OTUs**	**Singletons**	**Coverage**	**OTUsim**	**OTUsim_std**	**CompBias**	**#Reads passing filters**	
511	147	0.3932	85.7	10.1	0.1804	1460	
**SUB_1124**							
**OTUs**	**OTUs_std**	**Singletons**	**Singletons_std**	**Coverage**	**Coverage_std**	**OTUsim**	**OTUsim_std**
435.01	6.69	128.29	6.44	0.3776	0.3776	85.88	10.06

PyroNoise

**NoSUB**							
**OTUs**	**Singletons**	**Coverage**	**OTUsim**	**OTUsim_std**	**CompBias**	**#Reads passing filters**	
2457	1618	0.2246	83.41	8.51	0.1171	3625	
**SUB_1124**							
**OTUs**	**OTUs_std**	**Singletons**	**Singletons_std**	**Coverage**	**Coverage_std**	**OTUsim**	**OTUsim_std**
897.09	11.7	656.78	15.23	0.1450	0.1453	83.81	8.64

USEARCH expected errors

**NoSUB**							
**OTUs**	**Singletons**	**Coverage**	**OTUsim**	**OTUsim_std**	**CompBias**	**#Reads passing filters**	
689	223	0.3605	85.58	10.07	0.1753	1620	
**SUB_1124**							
**OTUs**	**OTUs_std**	**Singletons**	**Singletons_std**	**Coverage**	**Coverage_std**	**OTUsim**	**OTUsim_std**
537.47	8.24	186.19	7.76	0.3347	0.3348	85.78	10.01

Ion62

Poisson binomial filtering

**NoSUB**							
**OTUs**	**Singletons**	**Coverage**	**OTUsim**	**OTUsim_std**	**CompBias**	**#Reads passing filters**	
488	167	0.4049	85.79	10.5	0.1639	1556	
**SUB_1124**							
**OTUs**	**OTUs_std**	**Singletons**	**Singletons_std**	**Coverage**	**Coverage_std**	**OTUsim**	**OTUsim_std**
401.22	7.45	138.8	6.1	0.3897	0.3897	86.08	10.44

PyroNoise

**NoSUB**							
**OTUs**	**Singletons**	**Coverage**	**OTUsim**	**OTUsim_std**	**CompBias**	**#Reads passing filters**	
2339	1498	0.2784	84.51	8.79	0.1037	4105	
**SUB_1124**							
**OTUs**	**OTUs_std**	**Singletons**	**Singletons_std**	**Coverage**	**Coverage_std**	**OTUsim**	**OTUsim_std**
806.76	10.54	577.76	14.15	0.1943	0.1945	85.11	8.88

USEARCH expected errors

**NoSUB**							
**OTUs**	**Singletons**	**Coverage**	**OTUsim**	**OTUsim_std**	**CompBias**	**#Reads passing filters**	
664	274	0.3721	85.3	10.49	0.1500	1743	
**SUB_1124**							
**OTUs**	**OTUs_std**	**Singletons**	**Singletons_std**	**Coverage**	**Coverage_std**	**OTUsim**	**OTUsim_std**
492.7	9.27	209.19	9.24	0.3491	0.3492	85.66	10.46

Ion64

Poisson binomial filtering

NoSUB

OTUs	Singletons	Coverage	OTUsim	OTUsim_std	CompBias	#Reads passing filters
542	133	0.3689	85.32	10.77	0.1924	1339

SUB_1124

OTUs	OTUs_std	Singletons	Singletons_std	Coverage	Coverage_std	OTUsim	OTUsim_std
482.73	6.14	133.27	5.17	0.3557	0.3557	85.47	10.7

PyroNoise

NoSUB

OTUs	Singletons	Coverage	OTUsim	OTUsim_std	CompBias	#Reads passing filters
2405	1555	0.2196	83.94	8.59	0.1222	3491

SUB_1124

OTUs	OTUs_std	Singletons	Singletons_std	Coverage	Coverage_std	OTUsim	OTUsim_std
907.44	10.66	655.57	15.26	0.1396	0.1400	84.53	8.53

USEARCH expected errors

NoSUB

OTUs	Singletons	Coverage	OTUsim	OTUsim_std	CompBias	#Reads passing filters
744	228	0.3243	85.16	10.54	0.1651	1488

SUB_1124

OTUs	OTUs_std	Singletons	Singletons_std	Coverage	Coverage_std	OTUsim	OTUsim_std
606.01	7.98	212.37	8.51	0.3020	0.3021	85.4	10.45

Ion68

Poisson binomial filtering

NoSUB

OTUs	Singletons	Coverage	OTUsim	OTUsim_std	CompBias	#Reads passing filters
652	245	0.3514	86.62	9.58	0.1458	1430

SUB_1124

OTUs	OTUs_std	Singletons	Singletons_std	Coverage	Coverage_std	OTUsim	OTUsim_std
557.63	7.39	220.12	6.87	0.3330	0.3331	86.86	9.48

PyroNoise

NoSUB

OTUs	Singletons	Coverage	OTUsim	OTUsim_std	CompBias	#Reads passing filters
2436	1646	0.2323	84.79	8.13	0.0796	3672

SUB_1124

OTUs	OTUs_std	Singletons	Singletons_std	Coverage	Coverage_std	OTUsim	OTUsim_std
889.97	10.26	667.26	14.59	0.1485	0.1488	85.32	8.19

USEARCH expected errors

NoSUB

OTUs	Singletons	Coverage	OTUsim	OTUsim_std	CompBias	#Reads passing filters
897	379	0.3240	86.47	9.31	0.1581	1790

SUB_1124

OTUs	OTUs_std	Singletons	Singletons_std	Coverage	Coverage_std	OTUsim	OTUsim_std
642.5	10.33	288.57	11.29	0.2908	0.2909	86.83	9.21

Ion71

Poisson binomial filtering

NoSUB

OTUs	Singletons	Coverage	OTUsim	OTUsim_std	CompBias	#Reads passing filters
681	217	0.3744	84.84	10.37	0.1503	1740

SUB_1124

OTUs	OTUs_std	Singletons	Singletons_std	Coverage	Coverage_std	OTUsim	OTUsim_std
511.78	8.77	163.23	7.96	0.3440	0.3441	85.13	10.33

PyroNoise

NoSUB

OTUs	Singletons	Coverage	OTUsim	OTUsim_std	CompBias	#Reads passing filters
2658	1744	0.2157	83.35	9.05	0.0902	3910

SUB_1124

OTUs	OTUs_std	Singletons	Singletons_std	Coverage	Coverage_std	OTUsim	OTUsim_std
897.24	12.08	644.82	15.35	0.1413	0.1417	83.89	9.03

USEARCH expected errors

NoSUB

OTUs	Singletons	Coverage	OTUsim	OTUsim_std	CompBias	#Reads passing filters
880	313	0.3447	84.54	10.28	0.1447	1899

SUB_1124

OTUs	OTUs_std	Singletons	Singletons_std	Coverage	Coverage_std	OTUsim	OTUsim_std
612.22	10.41	232.17	9.63	0.3007	0.3008	84.83	10.21

Ion72

Poisson binomial filtering

NoSUB

OTUs	Singletons	Coverage	OTUsim	OTUsim_std	CompBias	#Reads passing filters
714	236	0.3415	86.5	9.44	0.1436	1461

SUB_1124

OTUs	OTUs_std	Singletons	Singletons_std	Coverage	Coverage_std	OTUsim	OTUsim_std
598.79	8.32	209.6	7.97	0.3201	0.3202	86.71	9.4

PyroNoise

NoSUB

OTUs	Singletons	Coverage	OTUsim	OTUsim_std	CompBias	#Reads passing filters
2692	1760	0.2086	84.92	8.48	0.0785	3804

SUB_1124

OTUs	OTUs_std	Singletons	Singletons_std	Coverage	Coverage_std	OTUsim	OTUsim_std
935.42	11.9	691.38	15.96	0.1250	0.1254	85.5	8.38

USEARCH expected errors

NoSUB

OTUs	Singletons	Coverage	OTUsim	OTUsim_std	CompBias	#Reads passing filters
936	338	0.3249	86.26	9.42	0.1247	1762

SUB_1124

OTUs	OTUs_std	Singletons	Singletons_std	Coverage	Coverage_std	OTUsim	OTUsim_std
685.38	9.27	272.34	8.96	0.2791	0.2792	86.64	9.31

Ion76

Poisson binomial filtering

NoSUB

OTUs	Singletons	Coverage	OTUsim	OTUsim_std	CompBias	#Reads passing filters
531	107	0.4090	85.97	9.89	0.1232	1637

SUB_1124

OTUs	OTUs_std	Singletons	Singletons_std	Coverage	Coverage_std	OTUsim	OTUsim_std
426.2	7.34	92.3	6.07	0.3870	0.3870	86.21	9.77

PyroNoise

NoSUB

OTUs	Singletons	Coverage	OTUsim	OTUsim_std	CompBias	#Reads passing filters
2448	1499	0.2423	83.7	8.29	0.0926	3793

SUB_1124

OTUs	OTUs_std	Singletons	Singletons_std	Coverage	Coverage_std	OTUsim	OTUsim_std
878.6	10.9	591.73	15.99	0.1601	0.1604	84.21	8.22

USEARCH expected errors

NoSUB

OTUs	Singletons	Coverage	OTUsim	OTUsim_std	CompBias	#Reads passing filters
679	165	0.3765	85.9	9.83	0.1126	1697

SUB_1124

OTUs	OTUs_std	Singletons	Singletons_std	Coverage	Coverage_std	OTUsim	OTUsim_std
522.18	8.14	138.67	6.45	0.3491	0.3492	86.17	9.74

Ion78

Poisson binomial filtering

NoSUB

OTUs	Singletons	Coverage	OTUsim	OTUsim_std	CompBias	#Reads passing filters
557	155	0.4028	86.82	9.6	0.1143	1728

SUB_1124

OTUs	OTUs_std	Singletons	Singletons_std	Coverage	Coverage_std	OTUsim	OTUsim_std
424.47	8.2	125.41	7.18	0.3768	0.3769	87.05	9.5

PyroNoise

NoSUB

OTUs	Singletons	Coverage	OTUsim	OTUsim_std	CompBias	#Reads passing filters
2383	1603	0.2305	84.11	8.21	0.0817	3668

SUB_1124

OTUs	OTUs_std	Singletons	Singletons_std	Coverage	Coverage_std	OTUsim	OTUsim_std
853.33	10.69	631.01	14.98	0.1609	0.1612	84.6	8.13

USEARCH expected errors

NoSUB

OTUs	Singletons	Coverage	OTUsim	OTUsim_std	CompBias	#Reads passing filters
717	241	0.3686	86.32	9.6	0.1284	1811

SUB_1124

OTUs	OTUs_std	Singletons	Singletons_std	Coverage	Coverage_std	OTUsim	OTUsim_std
517.68	9.58	184.03	7.33	0.3380	0.3381	86.66	9.44

Ion79

Poisson binomial filtering

NoSUB

OTUs	Singletons	Coverage	OTUsim	OTUsim_std	CompBias	#Reads passing filters
536	214	0.3891	89.34	7.45	0.1557	1501

SUB_1124

OTUs	OTUs_std	Singletons	Singletons_std	Coverage	Coverage_std	OTUsim	OTUsim_std
445.11	7.14	190.05	7.6	0.3714	0.3714	89.45	7.47

PyroNoise

NoSUB

OTUs	Singletons	Coverage	OTUsim	OTUsim_std	CompBias	#Reads passing filters
2447	1648	0.2847	87.14	6.9	0.0571	4353

SUB_1124

OTUs	OTUs_std	Singletons	Singletons_std	Coverage	Coverage_std	OTUsim	OTUsim_std
804.79	12.08	608.33	14.64	0.1924	0.1926	87.74	6.83

USEARCH expected errors

NoSUB

OTUs	Singletons	Coverage	OTUsim	OTUsim_std	CompBias	#Reads passing filters
698	294	0.3728	89.12	7.44	0.1551	1753

SUB_1124

OTUs	OTUs_std	Singletons	Singletons_std	Coverage	Coverage_std	OTUsim	OTUsim_std
519.08	7.58	237.14	8.44	0.3434	0.3434	89.34	7.47

Supplementary Note 6. Random removal of reads does not explain the taxonomic biases generated by the different filtering methods

Figure SN6.1. Random removal of reads does not explain the taxonomic biases generated by the different filtering methods

As demonstrated in **Supplementary Note SN2.1**, random removal of reads from a community will have a minor effect on the observed community composition. In order to exemplify this with experimental data, we have randomly removed an increasing number of reads from six mock and environmental communities sequenced with the three platforms analyzed in this study, and we have calculated the generated taxonomic bias (measured as the Bray-Curtis dissimilarity between the unfiltered and the subsampled communities, as described in the methods section). For each data point, ten independent subsamples were taken, and the average and standard deviation were reported. We have compared the resulting biases with those obtained when applying the different filtering algorithms. Taxonomic bias was calculated at the genus level, except for the 454 and IonTorrent environmental datasets (HumanOral, Ion49), in which it was calculated at the family level. **a)** 454. **b)** MiSeq. **c)** IonTorrent.

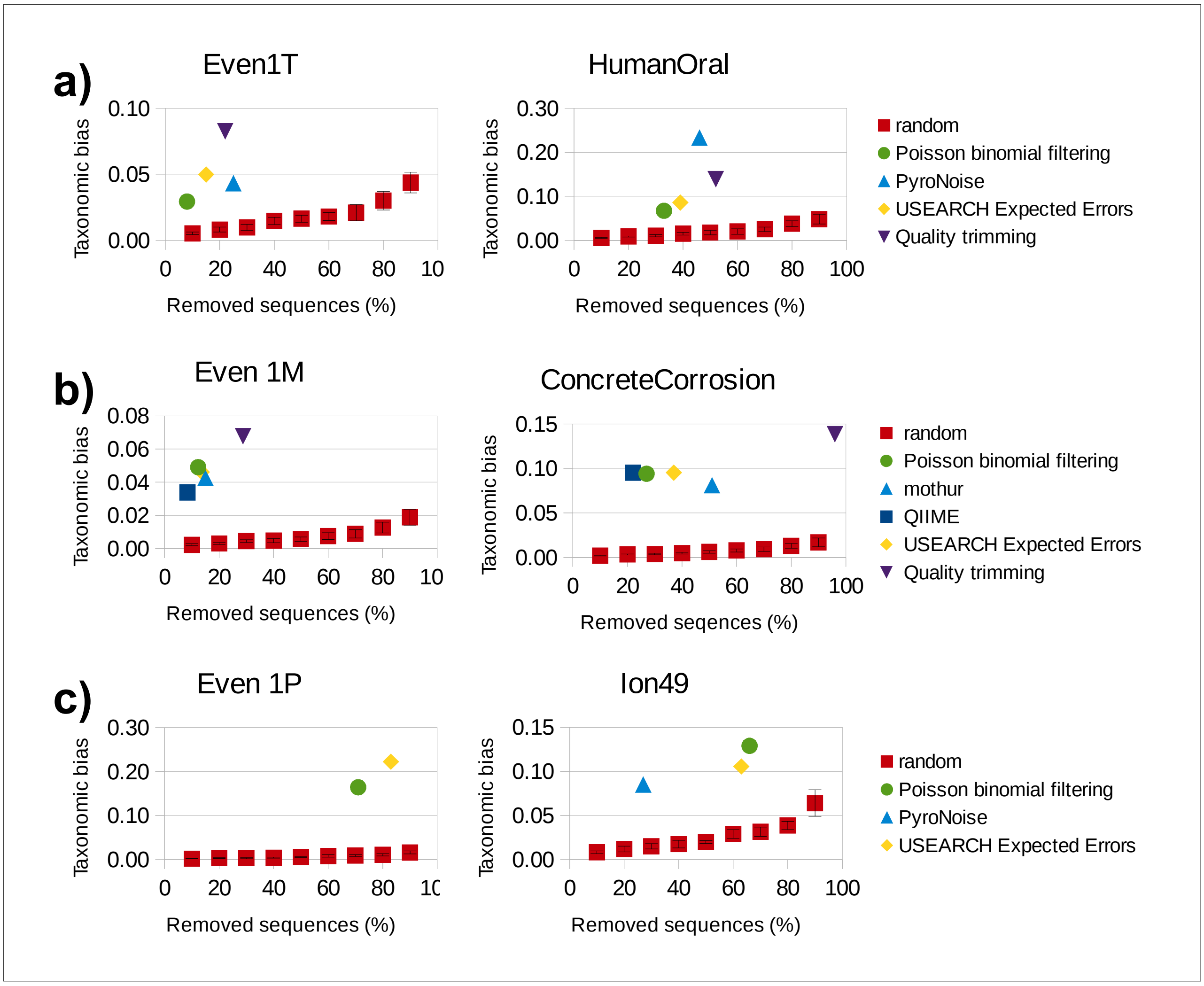

Supplementary Note 7. Effect of the choice of quality-filtering method on the inference of ecological patterns

While the previous sections (and previous works, see references in main test) show that the choice of filtering method can greatly affect features such as the number of discarded sequences, or the observed number of OTUs and singletons, molecular ecology studies are more likely to be focused on inter-sample comparisons. Therefore, analysing the same set of samples with two filtering methods could theoretically lead to the same ecological interpretation, even if the individual results for both methods greatly differ on a sample-by-sample basis. In this section we explore how the choice of filtering method can affect the inference of ecological patterns based on relative community richness, diversity and composition. The datasets used in this section were obtained from the mothur SOP for the analysis of 454, Illumina and IonTorrent datasets, and can be downloaded from the following url adresses:
http://www.mothur.org/wiki/454_SOP
http://www.mothur.org/wiki/MiSeq_SOP
http://www.mothur.org/wiki/Ion_Torrent_sequence_analysis_using_Mothur

Table SN7.1. Effect of the choice of filtering method on relative community richness

The datasets for the three sequencing platforms, obtained from their corresponding mothur SOP webpages, were processed using different filtering methods as described in **Supplementary Note 9**, and the Chao1 richness index was calculated for each sample and filtering method. This table shows the Pearson correlation of the Chao1 index profiles through all samples for each possible pair of filtering methods. In all cases, the filtered datasets were subsampled to a similar number of sequences per sample, in order to avoid size effects. Abbreviations: PBF, Poisson binomial filtering; UEE, USEARCH expected errors; QT, quality trimming.

454

	PyroNoise	QT	UEE
**PBF**	0.6952	0.8031	0.8954
**UEE**	0.8336	0.9080	
**QT**	0.8487		

Illumina

	mothur	QIIME	QT	UEE
**PBF**	0.7422	0.3230	0.5597	0.5398
**UEE**	0.2881	0.3622	0.4959	
**QT**	0.3958	0.1960		
**QIIME**	-0.0345			

IonTorrent

	PyroNoise	EE
**moira**	0.6380	0.9534
**EE**	0.5903	

Table SN7.2. Effect of the choice of filtering method on relative community diversity

The datasets for the three sequencing platforms, obtained from their corresponding mothur SOP webpages, were processed using different filtering methods as described in **Supplementary Note 9**, and the Shannon diversity index was calculated for each sample and filtering method. This table shows the Pearson correlation of the Shannon index profiles through all samples for each possible pair of filtering methods. In all cases, the filtered datasets were subsampled to a similar number of sequences per sample, in order to avoid size effects. Abbreviations: PBF, Poisson binomial filtering; UEE, USEARCH expected errors; QT, quality trimming.

454

	mothur	QIIME	QT	UEE
**PBF**	0.9879	0.9879	0.9892	0.9907
**UEE**	0.9902	0.9933	0.9889	
**QT**	0.9834	0.9902		
**QIIME**	0.9931			

Illumina

	PyroNoise	QT	UEE
**PBF**	0.9993	0.9980	0.9991
**UEE**	0.9989	0.9991	
**QT**	0.9983		

IonTorrent

	PyroNoise	EE
**moira**	0.5434	0.9801
**EE**	0.5786	

Table SN7.3. Effect of the choice of filtering method on relative community diversity

The datasets for the three sequencing platforms, obtained from their corresponding mothur SOP webpages, were processed using different filtering methods as described in **Supplementary Note 9**, and a Bray-Curtis distance matrix between all pairs of samples was calculated for each filtering method. This table shows the Pearson correlation of the inter-sample distance matrices for every possible pair of filtering methods. All the reported correlations were deemed to be significant (Mantel test, $p < 0.01$). In all cases, the filtered datasets were subsampled to a similar number of sequences per sample, in order to avoid size effects Abbreviations: PBF, Poisson binomial filtering; UEE, USEARCH expected errors; QT, quality trimming.

454

	mothur	QIIME	QT	UEE
**PBF**	0.9904	0.9914	0.9887	0.9905
**UEE**	0.9903	0.9900	0.9914	
**QT**	0.9913	0.9906		
**QIIME**	0.9869			

Illumina

	PyroNoise	QT	UEE
**PBF**	0.9996	0.9997	0.9996
**UEE**	0.9997	0.9997	
**QT**	0.9994		

IonTorrent

	PyroNoise	EE
**moira**	0.9230	0.9939
**EE**	0.9192	

Supplementary Note 8. Execution time of the Poisson binomial filtering algorithm and its Poisson approximation

Table SN8.1. Execution time of the Poisson binomial filtering algorithm and its Poisson approximation on 250 nt sequences

Simulated datasets were generated using the XS program (Pratas *et al.*, 2014). The script moira.py was run on an Intel Core i7-2670QM at 2.2 GHz, using a single core. Abbreviations: PB/CPython: Poisson binomial algorithm, naive python implementation run on the Cpython interpreter; PB/Cpython+C: Poisson binomial algorithm, python + C extension run on the Cpython interpreter; PB/Pypy: Poisson binomial algorithm, naive python implementation run on the PyPy interpreter; Poisson/Cpython: Poisson approximation, naive python implementation run on the Cpython interpreter; Poisson/PyPy: Poisson approximation, naive python implementation run on the PyPy interpreter.

#Sequences	PB / CPython (s)	PB / CPython + C (s)	PB / PyPy (s)	Poisson / CPython (s)	Poisson / PyPy (s)
1,000	25.1	0.3	3.1	0.2	0.3
5,000	121.9	1.7	15.2	1	1.1
10,000	247.4	3.3	29.6	2	1.2
50,000	1384.7	16.8	149.5	10.1	5.7
100,000	2413.8	33.2	320.6	20.2	11.4
500,000	11849.1	170.5	1575.4	105.7	58.7
1,000,000	23567.6	347.9	3031.9	209.1	119.3

Table SN8.2. Execution time of the Poisson binomial filtering algorithm and its Poisson approximation on 10000 sequences of variable length.

Simulated datasets were generated using the XS program (Pratas *et al.*, 2014). The script moira.py was run on an Intel Core i7-2670QM at 2.2 GHz, using a single core. Abbreviations: PB/CPython: Poisson binomial algorithm, naive python implementation run on the Cpython interpreter; PB/Cpython+C: Poisson binomial algorithm, python + C extension run on the Cpython interpreter; PB/Pypy: Poisson binomial algorithm, naive python implementation run on the PyPy interpreter; Poisson/Cpython: Poisson approximation, naive python implementation run on the Cpython interpreter; Poisson/PyPy: Poisson approximation, naive python implementation run on the PyPy interpreter.

Length (nt)	PB / CPython (s)	PB / CPython + C (s)	PB / PyPy (s)	Poisson / CPython (s)	Poisson / PyPy (s)
100	35.4	0.9	5.3	1	1.2
200	148.7	2.2	18.4	1.7	1.3
300	348.9	4.5	41.9	2.2	1.3
400	689.1	8.7	78.6	2.9	1.6
500	1179.1	13.6	132.2	3.5	1.8
600	1836.7	20.3	212.8	4.3	2.1
700	2648.6	29.6	288.7	5	2.2
800	3668.8	40.4	429.8	5.7	2.4

Supplementary Note 9. Theoretical and practical differences between the Poisson binomial filtering algorithm and USEARCH expected errors filtering

During the review process of this manuscript, new information regarding the USEARCH expected errors (E_max) filtering algorithm was published by Edgar & Flyvbjerg (2015). They calculate the expected errors of a sequence by using a Poisson distribution. This has the advantage of being extremely simple, as the expected errors of a sequence can be calculated from a Poisson distribution as the sum of the error probabilities of each base. However, we have shown that approximating a Poisson binomial distribution to a Poisson distribution may lead to significant differences in practice, as even one bad-quality base in an otherwise good-quality sequence will result in approximation errors according to Le Cam's theorem (**Supplementary Note SN5.5**).

The USEARCH expected errors filtering is therefore different to the Poisson binomial filtering algorithm presented in this work, but equivalent to its Poisson approximation, as shown by Edgar & Flyvbjerg in Eq. (6) of their paper. Even in that case, we believe that the use of the predicted maximum errors j_{ξ} presented in our work could be in a sense more clear than Edgar & Flyvbjerg's E/E_max, since the term E_max (and our own term maximum predicted errors) could suggest that an upper bound is being placed on the number of errors, that is, that no sequence passing the filter should have more than E_max (or j_{tol}, as per our notation) errors.

The relationship between the expected errors E and the probability of having less than E errors is non-trivial. In **Supplementary Figure SN9.1** we show the fraction of sequences that, being E < 1 (and therefore being accepted as correct by the E_max method), would indeed have 0 true errors, assuming a Poisson distribution. For very low values of E, almost all sequences are classified correctly, but for E close to 1 less than 40% of the sequences that are accepted as having zero errors would really have zero errors, leading to a large underestimation of the number of errors.

In order to test the impact of this issue, we have validated both methods by classifying the sequences of the Even1P dataset, as described in the methods section of the main text ("Validation of Poisson binomial filtering on Mock Community data"). The flag --eeout was provided to USEARCH in order to obtain the expected errors of each sequence. Parameters were adjusted so that both methods discarded approximately the same number of sequences. **Supplementary Figure SN9.2** shows that, for a similar number of discarded sequences, USEARCH produced far more false negatives (that is, sequences with E < E_max that nonetheless had more than E_max true errors) than PBF.

Even a small proportion of erroneous sequences could significantly affect the results of 16S-rRNA gene-based molecular ecology studies. In this context, the users will want to establish a clear upper bound for the number of errors present in their quality-filtered sequences. The error numbers reported by PBF can be easily interpreted by the user: the sequence has a fixed (but user-settable) probability of having more errors than reported. On the other hand, an E_max filter will let pass many sequences with more than E_max errors, in a proportion that is not fixed but depends on E_max. This makes the use of mock/PhiX data to correctly set the E_max parameter a critical step, as described in Edgar & Flyvbjerg (2015). PBF, or its Poisson approximation (as used in this work), is free of this

particular issue.

In any case, we want to note that Edgar & Flyvbjerg recommend the use of mock/PhiX data to measure the correlation of Q-score derived benchmarks to the true error rate of the sequences. While Q-scores were generally a good predictor of the true error rates for all the mock datasets analysed in this study (which covered 454, Illumina and MiSeq platforms), we fully agree with Edgar & Flyvbjerg (and others) in that the use of mock/PhiX data to fine tune the filtering parameters will result in a more accurate quality-filtering.

Figure SN9.1. Probability of a sequence having zero errors for different values of E.

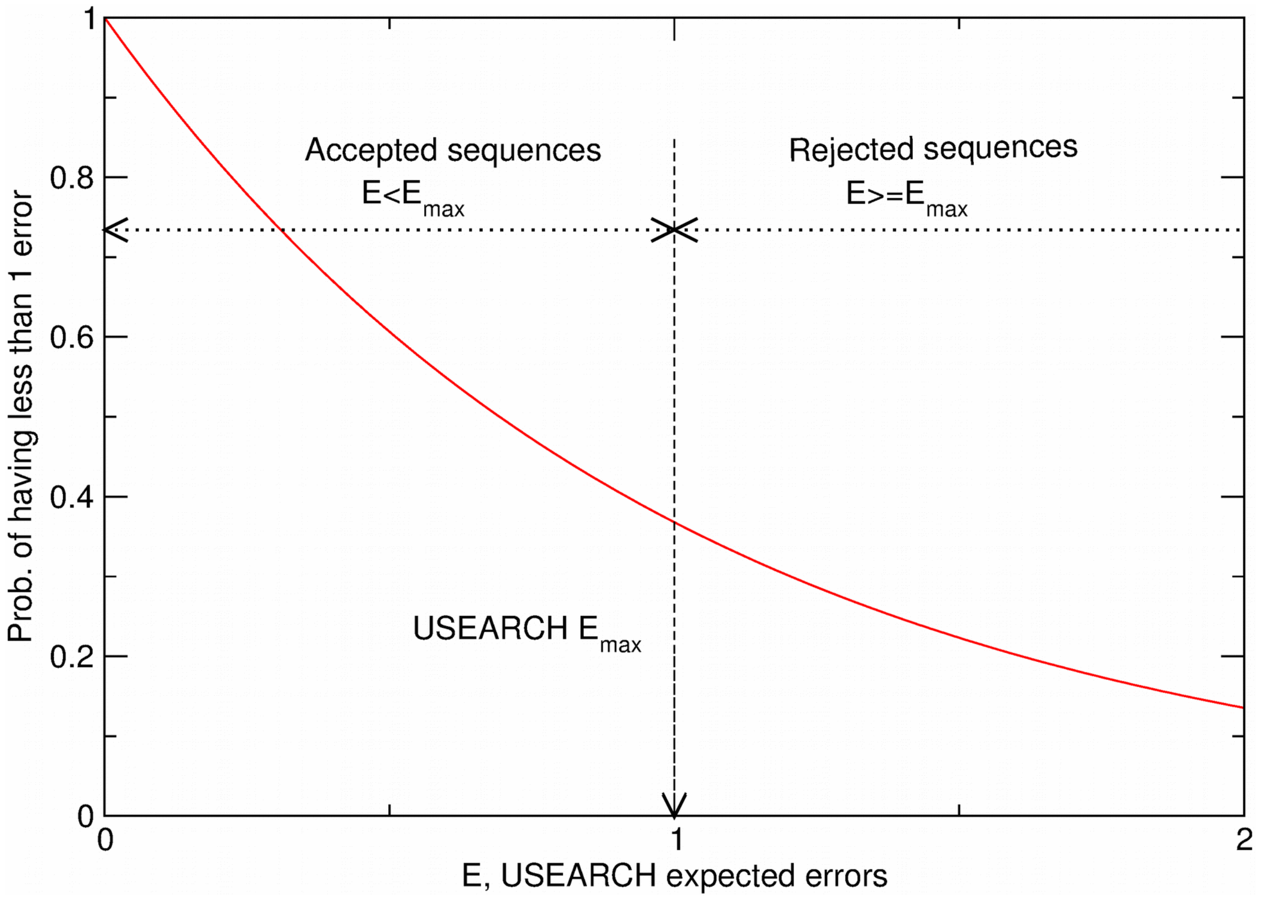

Figure SN9.2. Comparison between the number of errors j_ξ and E predicted by the Poisson binomial and USEARCH filter (with and without the previous application of the collapsing step presented in this work) algorithms, and the true number of errors for all sequences from the Even1P mock community dataset. Dots represent unique sequences. True mock community sequences are plotted in blue, contaminant sequences are plotted in gray, and chimeric sequences are plotted in red. The blue background represents sequence abundance (note that for PBF and USEARCH-collapsed few unique sequences may have a high number of representatives, and vice versa). Red lines indicate the error cut-off (j_{tol}), which was adjusted so that all the methods retained approximately the same number of sequences (42-44%). The plot is thus divided in four quadrants corresponding to correctly retained sequences (lower left), correctly discarded sequences (upper right), incorrectly discarded sequences (upper left) and incorrectly retained sequences (lower right). The percentage of true mock community sequences present on each quadrant is also indicated.

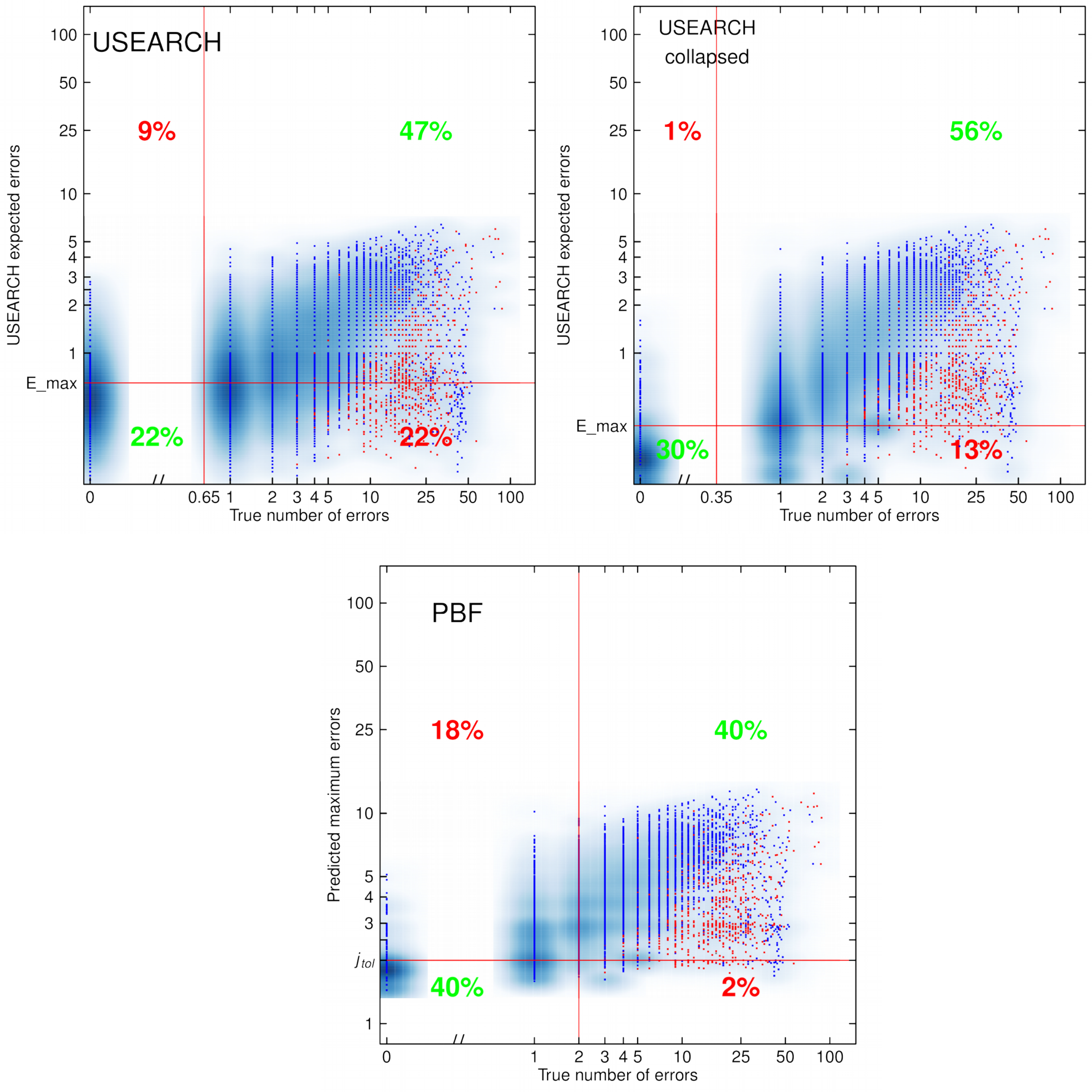

Supplementary Note 10. Software commands, parameters and computational resources

SN10.1 Primer removal from mock and environmental datasets

Some of the environmental datasets analyzed in this study had missing information regarding the PCR primers that were used for 16S amplification. In order to account for this issue, putative primers were predicted for all the environmental datasets using the *tagcleaner.pl* script (Schmieder *et al.*, 2010). In all the 454 environmental datasets, a putative primer was identified with more than 99% certainty. However, *tagcleaner.pl* failed to clearly identify primer sequences in the environmental Illumina datasets and in the mock IonTorrent datasets. While this probably meant that the authors removed primer sequences before uploading their libraries to the NCBI Sequence Read Archive, we trimmed 20 nucleotides from the 5' end of both the forward and reverse reads of those datasets in order to minimize the risk of including primer sequences into the analysis. As for the Illumina mock community datasets, primers and adaptors had been removed from the libraries beforehand. Therefore, no primer removal step was introduced in the Illumina mock community processing pipelines.

SN10.2 Commands used for filtering the 454 datasets

SN 10.2.1 Commands for Pyro-Noise

```
mothur
sffinfo(sff=reads.sff)
trim.flows(flow=reads.flow, oligos=primers.txt, pdiffs=2)
shhh.flows(file=reads.flow.files)
trim.seqs(fasta=reads.trim.shhh.fasta, name=reads.trim.shhh.names,
oligos=primers.txt, pdiffs=2, bdiffs=1, maxhomop=8, minlength=250,
keepfirst=250, flip=T, processors=2)
quit()
```

SN 10.2.2 Commands for Quality-trimming

```
mothur
trim.seqs(fasta=reads.fasta, qfile=reads.qual, oligos=primers.txt,
pdiffs=2, bdiffs=1, maxhomop=8)
make.fastq(fasta=reads.trim.fasta, qfile=reads.trim.qual)
quit()

usearch -fastq_filter reads.trim.fastq -fastaout filtered.fasta
-fastq_truncqual 15 -fastq_trunclen 250
```

SN 10.2.3 Commands for USEARCH expected errors

```
mothur
trim.seqs(fasta=reads.fasta, qfile=reads.qual, oligos=primers.txt,
pdiffs=2, bdiffs=1, maxhomop=8)
make.fastq(fasta=reads.trim.fasta, qfile=reads.trim.qual)
quit()

usearch -fastq_filter reads.trim.fastq -fastaout filtered.fasta
-fastq_maxeee 0.5 -fastq_trunclen 250
```

SN 10.2.4 Commands for Poisson binomial filtering

```
mothur
trim.seqs(fasta=reads.fasta, qfile=reads.qual, oligos=primers.txt,
pdiffs=2, bdiffs=1, maxhomop=8)
quit()

moira.py --forward_fasta reads.trim.fasta --forward_qual
reads.trim.qual --truncate 250 --uncert 0.01 --alpha 0.005
```

SN10.3 Commands used for filtering the Illumina datasets

SN 10.3.1 Length truncation of contigs generated from Illumina paired-end reads

While the sequences used in this study were normally truncated to 250 nucleotides, some environmental communities were sequenced with pairs of primers that generated smaller contigs. In those cases, contigs were truncated to a smaller length. This modified the values of the following parameters:

- mothur: *minlength, keepfirst*
- Quality-trimming and USEARCH expected errors: *fastq_trunclen*
- Poisson binomial filtering: *truncate*

A file containing the truncation lengths used for those datasets is provided as a **Supplementary resource.**

SN 10.3.2 Commands for mothur

NOTE: mothur developers recommended a value of 275 for the maxlength parameter when analyzing the V4 region of the 16S rRNA gene. Some of the environmental communities were sequenced using different primers. For them, a custom maxlength value was selected, based on the contig length distribution, in order to discard the clearly missasembled contigs. A file containing the maxlength values used in these datasets is provided as a **Supplementary resource.**

```
mothur
make.contigs(ffasta=reads_1.fasta, rfasta=reads_2.fasta,
fqfile=reads_1.qual, rqfile=reads_2.qual)
trim.seqs(fasta=reads.trim.contigs.fasta, maxambig=0,
maxlength=275, minlength=250, keepfirst=250)
quit()
```

SN 10.3.3 Commands for QIIME

```
join_paired_ends.py -f reads_1.fastq -r reads_2.fastq -o merged
split_libraries_fastq.py -i merged/fastqjoin.join.fastq -m
primer_map.txt -o filtered --barcode_type=not-barcoded
--sample_ids=sample_name -r 3 -p 0.75 -q 3 -n 0 –phred_offset=33
```

SN 10.3.4 Commands for Quality-trimming

```
usearch -fastq_mergepairs reads_1.fastq -reverse reads_2.fastq
-fastq_truncqual 2 -fastqout merged.fastq
usearch -fastq_filter merged.fastq -fastaout filtered.fasta
-fastq_truncqual 15 -fastq_trunclen 250
```

SN 10.3.5 Commands for USEARCH expected errors

```
usearch -fastq_mergepairs reads_1.fastq -reverse reads_2.fastq
-fastq_truncqual 2 -fastqout merged.fastq
usearch -fastq_filter merged.fastq -fastaout filtered.fasta
-fastq_maxee 0.5 -fastq_trunclen 250
```

SN 10.3.6 Commands for Poisson binomial filtering

```
moira.py --paired --forward_fasta reads_1.fasta --forward_qual
reads_1.qual --reverse_fasta reads_2.fasta –reverse_qual
reads_2.qual --truncate 250 --uncert 0.01 --alpha 0.005
```

SN10.4 Commands used for filtering the IonTorrent datasets

SN 10.4.1 Commands for Pyro-Noise

```
mothur
sffinfo(sff=reads.sff)
trim.flows(flow=reads.flow, oligos=primers.txt, pdiffs=2)
shhh.flows(file=reads.flow.files, order=I)
trim.seqs(fasta=reads.trim.shhh.fasta, name=reads.trim.shhh.names,
oligos=primers.txt, pdiffs=1, bdiffs=1, maxhomop=8, maxambigs=0,
minlength=200, keepfirst=200, flip=T, processors=2)
quit()
```

SN 10.4.2 Commands for USEARCH expected errors

```
mothur
trim.seqs(fasta=reads.fasta, qfile=reads.qual, oligos=primers.txt,
pdiffs=1, bdiffs=1, maxhomop=8, maxambigs=0)
make.fastq(fasta=reads.trim.fasta, qfile=reads.trim.qual)
quit()

usearch -fastq_filter reads.trim.fastq -fastaout filtered.fasta
-fastq_maxeee 0.5 -fastq_trunclen 200
```

SN 10.4.3 Commands for Poisson binomial filtering

```
mothur
trim.seqs(fasta=reads.fasta, qfile=reads.qual, oligos=primers.txt,
pdiffs=1, bdiffs=1, maxhomop=8, maxambigs=0)
quit()

moira.py --forward_fasta reads.trim.fasta --forward_qual
reads.trim.qual --truncate 200 --uncert 0.01 --alpha 0.005
```

SN10.5 Common downstream processing pipeline

In order to evaluate the different methods on equal grounds, filtered reads were processed with a common downstream pipeline that included chimera-filtering with UCHIME (main text ref. 15), sample size standardization and OTU clustering. Reference databases were obtained from the mothur webpage. For each sample, the number of reads kept by the most stringent method was chosen as the sub-sampling size for the rest of the methods. Steps from 11 to 17 (for mock community datasets) or from 11 to 16 (for environmental community datasets) were repeated 100 times, and the results were retrieved and averaged with an in-house script. The script *moira.py* and mothur's command *shhh.flows* generated a mothur name file in addition to the filtered fasta file. For those two methods, step 1 was omitted. For the IonTorrent environmental datasets, step 3 parameters was substituted by "screen.seqs(fasta=filtered.unique.align, name=filtered.names, start, 1044, optimize=end)".

```
1)unique.seqs(fasta=filtered.fasta)

2)align.seqs(fasta=filtered.unique.fasta,
reference=silva.bacteria.fasta)

3)screen.seqs(fasta=filtered.unique.align, name=filtered.names,
optimize=start-end, criteria=95)

4)filter.seqs(fasta=filtered.unique.good.align, vertical=T,
trump=.)

5)unique.seqs(fasta=filtered.unique.good.filter.fasta,
name=filtered.good.names)

6)pre.cluster(fasta=filtered.unique.good.filter.unique.fasta,
name=filtered.unique.good.filter.names, diffs=2)

7)chimera.uchime(fasta=filtered.unique.good.filter.unique.preclust
er.fasta,
name=filtered.unique.good.filter.unique.precluster.names)

8)remove.seqs(fasta=filtered.unique.good.filter.unique.precluster.
fasta, name=filtered.unique.good.filter.unique.precluster.names)

9)classify.seqs(fasta=filtered.unique.good.filter.unique.precluste
r.pick.fasta,
name=filtered.unique.good.filter.unique.precluster.pick.names,
template=trainset9_032012.pds.fasta,
taxonomy=trainset9_032012.pds.tax, cutoff=40)

10)remove.lineage(fasta=filtered.unique.good.filter.unique.preclus
```

```
ter.pick.fasta,
name=filtered.unique.good.filter.unique.precluster.pick.names,
taxonomy=filtered.unique.good.filter.unique.precluster.pick.pds.wa
ng.taxonomy, taxon=Mitochondria-Chloroplast-Archaea-Eukaryota-
unknown)


11)sub.sample(fasta=filtered.unique.good.filter.unique.precluster.
pick.pick.fasta,
name=filtered.unique.good.filter.unique.precluster.pick.pick.names
, size=subsampling_size)

12)dist.seqs(fasta=filtered.unique.good.filter.unique.precluster.p
ick.pick.subsample.fasta, cutoff=0.15)

13)cluster(column=filtered.unique.good.filter.unique.precluster.pi
ck.pick.subsample.dist,
name=filtered.unique.good.filter.unique.precluster.pick.pick.subsa
mple.names)

14)get.oturep(fasta=filtered.unique.good.filter.unique.precluster.
pick.pick.subsample.fasta,
name=filtered.unique.good.filter.unique.precluster.pick.pick.subsa
mple.names,
list=filtered.unique.good.filter.unique.precluster.pick.pick.subsa
mple.an.list,
column=filtered.unique.good.filter.unique.precluster.pick.pick.sub
sample.dist, label=0.03)

16)align.seqs(fasta=filtered.unique.good.filter.unique.precluster.
pick.pick.subsample.an.0.03.rep.fasta,
reference=silva.bacteria.fasta)

17)align.seqs(fasta=filtered.unique.good.filter.unique.precluster.
pick.pick.subsample.an.0.03.rep.fasta, reference=HMP_mock.fasta)
```

SN10.6 Computational resources

Unless otherwise specified, all analyses were conducted on the Data Intensive Academic Grid (DIAG) computing infrastructure (http://diagcomputing.org/), which is funded by the National Science Foundation's MRI-R2 project #DBI-0959894.

Supplementary references

Bokulich N, Subramanian S, Faith JJ, Gevers D, Gordon, JI, Knight R. *et al.* (2013). Quality-filtering vastly improves diversity estimates from Illumina amplicon sequencing. *Nat. Methods* **10**: 57-59.

Brockman W, Alvarez P, Young S, Garber M, Giannoukos G, Lee WL, Russ C, Lander ES, Nusbaum C, Jaffe DB (2008). Quality scores and SNP detection in sequencing-by-synthesis systems. *Genome Res*, **18**: 763-770.

Butler K & Stephens M (1993) The distribution of a sum of binomial random variables. *Technical Report* **467**, Stanford University, Stanford, California, USA.

Cramér, H., Mathematical Models of Statistics (1999), Princeton University Press.

Gaspar J & Thomas W. (2013). Assessing the consequences of denoising marker-based metagenomic data. *PLOS One* **8**: e60458.

Haas BJ, Gevers D, Earl AM, Feldgarden M, Ward DV, Giannoukos G. (2011). Chimeric 16S rRNA sequence formation and detection in Sanger and 454-pyrosequenced PCR amplicons. *Genome Res.* **21**: 494-504.

Huber J, Morrison HG, Huse SM, Neal PR, Sogin ML, Mark Welch DB (2009) Effect of PCR amplicon size on assessments of clone library microbial diversity and community structure. *Environ. Microbiol*. **11**: 1292– 1302.

Klindworth A, Pruesse E, Schweer T, Peplies J, Quast C, Horn M, Glöckner FO (2013). Evaluation of general 16S ribosomal RNA gene PCR primers for classical and next generation sequencing-based diversity studies. *Nucleic Acids Res.* **41**: e1.

Kumar P, Brooker M, Dowd S & Camerlengo T (2011) Target region selection is a critical determinant of community fingerprints generated by 16S pyrosequencing. *PLOS One* **6**: e20956.

Edgar R, Haas B, Clemente J, Quince C & Knight R. (2011). UCHIME improves sensitivity and speed of chimera detection*. Bioinformatics* **27**: 2194-2200.

Edgar, R. UPARSE: highly accurate OTU sequences from microbial amplicon reads. (2013) *Nat. Methods.* **10**: 996–998.

Edgar R, & Flyvbjerg H (2015). Error filtering, pair assembly and error correction for next-

generation sequencing reads. *Bioinformatics*, btv401.

Poisson, S. D., Recherches sur la probabilité des jugements en matière criminelle et en matière civile (1837). Bachelier, Paris.

Polz M & Cavanaugh C. (1998). Bias in template-to-product ratios in multitemplate PCR. Appl. Environ. Microbiol. 64, 3724-3730.

Pratas D, Pinho AJ, & Rodrigues JM (2014). XS: a FASTQ read simulator. *BMC research notes*, **7**, 40.

Quast C, Pruesse E, Yilmaz P, Gerken J, Schweer T, Yarza P *et al.* (2013). The SILVA ribosomal RNA gene database project: improved data processing and web-based tools*. Nucl. Acids Res.* **41**: 590-596.

Quince C, Lanzén A, Curtis TP, Davenport RJ, Hall N, Head IM *et al.* (2009). Accurate determination of microbial diversity from 454 pyrosequencing data. *Nat. Methods* **6**: 639-641.

Ross M, Russ C, Costello M, Hollinger A, Lennon NJ, Hegarty R, Nusbaum C, Jaffe DB (2013). Characterizing and measuring bias in sequence data. *Genome Biol.* **29**: R51.

Salipante SJ, Kawashima T, Rosenthal C, Hoogestraat DR, Cummings LA, Sengupta DJ, Harkins TT, Cookson BT, Hoffman NG (2014). Performance Comparison of Illumina and Ion Torrent Next-Generation Sequencing Platforms for 16S rRNA-Based Bacterial Community Profiling. *Appl Environ Microbiol* **80**, 7583-7591.

Schloss P, Westcott SL, Ryabin T, Hall JR, Hartmann M, Hollister EB *et al.* (2009). Introducing mothur: open-source, platform-independent, community-supported software for describing and comparing microbial communities. *Appl. Environ. Microbiol.* **75**: 7537-7521.

Schmieder R, Lim YW, Rohwer F, Edwards R (2010). TagCleaner: Identification and removal of tag sequences from genomic and metagenomic datasets. *BMC Bioinformatics* **11**: 341.
